# Low Band Gap Fused Bicyclic Polymers with Heteroatoms Se and Te: A DFT-PBC Study

**DOI:** 10.1021/acsomega.5c04257

**Published:** 2026-03-02

**Authors:** Zeki Büyükmumcu, Fatma Selampinar

**Affiliations:** † Department of Chemistry, Faculty of Sciences, Erciyes University, Kayseri 38039, Turkey; ‡ Department of Chemistry, 7712 University of Connecticut, Storrs, Connecticut 06269-3060, United States

## Abstract

Designing low band gap conjugated polymers is critical for the development of advanced materials in organic electronics. This study focuses on DFT-PBC analysis of a series of bicyclic fused polymers containing heteroatoms Se and Te, using the hybrid functional B3PW91. The polymer geometries defined by cells containing two monomers connected in different configurations were initially optimized with an assumption that all the atoms are on the same plane due to interchain interactions in the solid state. Subsequently, the structures were optimized without these restrictions, starting from the nearly planar geometry, as small deviations were anticipated due to insufficient interchain interactions required for planar geometry. According to the band structure calculations, the band gap value for the planar structure with 4–6 connection positions, where two Se atoms occupy both heteroatom positions, was found to be 0.779 eV, which is very close to the experimentally determined value of 0.76 eV (Patra 11). The finding indicates that polymerization primarily occurs through this connection within theoretical limits. The band gap values for other structures with the same connection positions but different heteroatom pairs (Se–Te, Te–Se, and Te–Te) were also low, at 0.905, 0.745, and 0.730 eV, respectively. Conducting properties of the title polymers were assessed by comparing band gaps, bandwidth, and effective mass values. In conclusion, the planar structures of the polymers with 4–6 and 2–4 connections exhibit bandwidths comparable to those of polypyrrole and polythiophene, with their effective masses that are either improved or comparable with these benchmark materials. Furthermore, atomic and subshell compositions of the frontier orbitals were analyzed to gain insight into the variation of the band gap as a function of the heteroatom.

## Graphical abstract

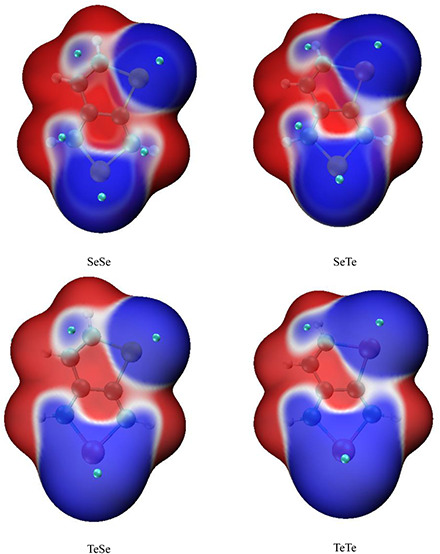

## 1 Introduction

Since the discovery of electrical conductivity in conjugated polymers as a result of doping,^1^ these materials have attracted significant interest in both basic scientific research and technological applications.^1^ Due to their lightweight, mechanical flexibility, and cost-effective production, materials containing conjugated polymers have been developed for applications such as light-emitting diodes, field-effect transistors, solar cells, electrochromic display devices, and different sensors.^2^

An essential set of parameters for material applications of semiconductors includes the energy levels of the valence and conduction bands and the energy gap, *E*_g_, between them. In the literature on organic semiconductors, these bands are generally referred to as HOMO and LUMO. Semiconducting polymers have the advantage of tuning the band gap and the energy levels of HOMO and LUMO by means of minor structural modifications. Such changes can significantly influence their electrical and optical properties.^2^

The parameters that determine the band gap of a polymer can be used to design structures with the desired band gaps. The band gap value has been determined by bond length alternation (BLA), planarity, resonance energy, substituents, intermolecular interaction, π-conjugation length, donor–acceptor structure, and heteroatom effects.^2–4^

Several definitions of the energy gap of semiconducting polymers have been proposed.^2,5^ According to Pomerantz, polymers with a gap lower than 1.5 eV are considered low band gap polymers, using the band gap of polyacetylene as a reference point.^6^ Low band gap polymers are of significant interest as optically transparent conductors since the shift of the absorption spectrum moves out of the visible region and into the near-infrared. In addition to the parameters mentioned above, fusing heterocycles to obtain monomers for polymerization is one of the most promising ways to obtain polymers with low energy gaps.^7^ Numerous polymers with fused heterocycles have been synthesized and theoretically studied.^7–15^ Among them, due to its relatively high optical transparency and high stability in the conducting state, poly­(ethylenedioxythiophene) (PEDOT) finds wide applications with a band gap value slightly greater than the threshold mentioned above.^7,10,16^ Another low band gap polymer, poly­(2-decylthieno­[3,4-*b*]­thiophene-4,6-diyl), which consists of a thiophene ring fused to another thiophene, shows a band gap of 0.92 eV.^12^ Its unsubstituted form, poly­(thieno­[3,4-*b*]­thiophene) was also synthesized and found to have a band gap value of 0.85 eV.^13^ Another polymer, poly­(thieno­[3,4-*b*]­furan), obtained by replacing one of the thiophene rings with a furan ring, has a band gap value of 1.03 eV and appears pale blue in its neutral form and a more transparent pale blue in the oxidized conducting state.^7^ The structures of poly­(thieno­[3,4-*b*]­furan), formed via different connection positions, were also analyzed theoretically by DFT in the same study. In addition, Patra et al. synthesized a series of new low-band gap thieno- or selenolo-fused polyselenophenes and selenolo-fused polythiophenes. By varying the combination of selenium and sulfur atoms within the poly­(thieno­[3,4-*b*]­furan) skeleton, they achieved band gap values between approximately 0.7 and 1.0 eV.^15^

Selenophene exhibits physical and chemical properties similar to those of thiophene. However, selenophene offers several advantages over thiophene in organic electronic applications, such as increased conductivity. This can be attributed to the Se···Se interactions, which cause a wider bandwidth in organic conductors and consequently facilitate interchain charge transfer.^17,18^ The aromatic character of the five-membered heterocyclic compounds decreases with increasing atomic radius. Large atoms, such as selenium and tellurium, cause a decrease in aromaticity.^19^ The band gap of heterocyclic polymers strongly depends on the type of heteroatom, which, in turn, affects both aromaticity and electronic properties.

Some properties of the Te atom are different from those of Se and S. The electronegativity of S is 2.58 and that of Se is 2.55, respectively, whereas Te has a much lower value of 2.10.^20^ In addition to its lower electronegativity, Te shows polarizability significantly higher than that of S and Se. Compared to thiophenes, tellurophenes and selenophenes have been found to have lower HOMO–LUMO gaps; the band gap of PTe is expected to be lower than those of PSe and PTh.^20^

In addition to the studies mentioned above, polymers containing Se and Te have been extensively investigated for various scientific and technological purposes.^21–31^ Sugimoto et al.^21^ conducted research on polyselenophene and polytellurophene, in which polytellurophene was chemically synthesized for the first time. Park et al.^22^ synthesized a tellurophene-containing low band gap polymer that absorbs light at longer wavelengths and exhibited a smaller band gap than its thiophene analogue. As a result of the DFT calculations, they concluded that the atomic substitution of sulfur with tellurium increased electronic coupling, thereby decreasing the length of inter-ring carbon–carbon bonds, causing a red shift in absorption. Nishiyama et al. prepared a tellurophene-containing π-conjugated polymer with fully coplanar ring units.^23^ As a result of various studies, it is expected that polymers containing tellurophene rings possess many interesting optoelectronic properties^23^ It has also been shown that tuning the optoelectronic properties of polymers could be achieved via controlled atom substitution in polymers containing five-membered chalcogenophene rings with S, Se, and Te.^24^

This study aims to analyze fused structures formed by different bicyclic combinations of selenophene and tellurophene rings. These monomers were previously studied as isolated molecules.^32–34^ The polymer derived from a monomer consisting of two selenophene rings, poly­(selenolo­[3,4-*b*]­selenophene), was synthesized, and its most stable isomer was briefly analyzed by Patra et al.^15^ Possible isomers of poly­(thieno­[3,4-*b*]­thiophene) by the oligomer approach^35^ and poly­(thieno­[3,4-*b*]­furan) by the PBC (Periodic Boundary Conditions) approach^7^ were studied employing DFT. In this study, we examine all the possible isomers formed via four different connection position combinations of four monomers. We compute and analyze geometric and electronic properties, which are helpful in predicting their conductive and optoelectronic properties.

## 2 Computational Methods

The polymers obtained from four distinct monomers, via four different connection position pairs, were optimized using DFT within the Periodic Boundary Conditions.^36^ In this way, a one-dimensional unit cell containing two monomers was defined to model the infinitely long polymer chains. The most suitable DFT functional for accurately predicting the band gap in good agreement with experimental values was identified as B3PW91,^37^ which has been shown to provide reliable results for organic polymers under typical experimental conditions.^38^ This function was employed with the standard basis set 6-31G­(d,p) for C and H, and the LanL2DZ basis set with effective core potentials for Se and Te.^39^ Geometry optimizations were performed without restriction with the assumption that all the atoms lie in the same plane due to interchain interactions within the solid-state phase. All calculations were carried out using the Gaussian 09 and 16 quantum chemistry packages.^40^ Band structure analyses were done on the optimized geometries using Gaussian, with additional analyses performed using Multiwfn 3.8 (dev).^41^ Initial geometries for optimization were sketched by GaussView 5.^42^ Data visualization was done by using GaussView 5,^42^ VMD,^43^ Avogadro,^44^ and Multiwfn 3.8 (dev).^41^

## 3 Results and Discussion

### 3.1 Reactivity of C Atoms at Different Positions

The general structure of the monomer, with two positions (X and Y) defined for the heteroatoms as described in reference 17, is shown in Figure 1. Monomers containing heteroatoms are abbreviated as XY throughout the text. (In Figure 1, Te is in the X position, and Se is in the Y position. Therefore, this structure is abbreviated as TeSe.) Nomenclature rules for numbering atoms were not applied to maintain consistency for comparisons based on heteroatom positions. Four open positions with C atoms are available to form chemical bonds with another monomer. The numbering of these positions is also shown in the same figure.

**Figure 1:**
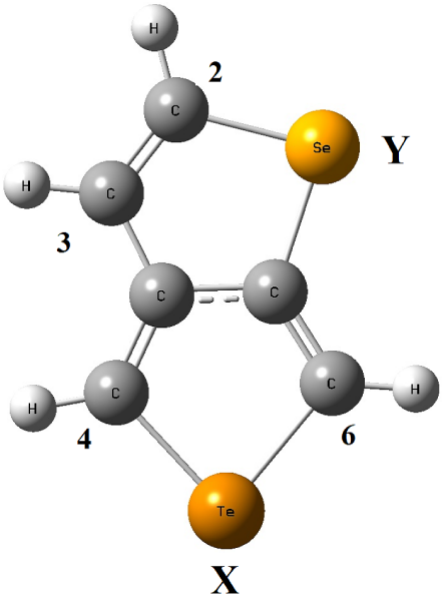
General structure of the monomer with two positions, X and Y, defined for heteroatoms and numbering of C positions, which are open to make bonds. (Te is in the X position, Se is in the Y position. Therefore, this structure is abbreviated as TeSe).

The electrochemical polymerization of heterocycles, such as thiophene^45^ and selenophene,^46^ has been studied, with the findings indicating that radical–radical couplings are the more likely mechanism. Therefore, we first investigated the reactive sites for radical attacks. The unpaired electron density of atoms in a cation radical was chosen as a suitable reactivity parameter.^47^ The spin density can be obtained experimentally by electron spin resonance (ESR) spectrometry or calculated through quantum chemical methods.^48^ Reactivity–structure correlations for the electropolymerization of pyrrole were studied by calculating the spin density distribution of radical cations employing INDO/CNDO methods.^48^ The spin distribution of the thieno­[3,4-*b*]­furan radical was calculated to predict reactive sites, and the highest spin distributions were obtained for the 4 and 6 positions.^7^

Mulliken atomic spin densities of cationic radicals of the title monomers are listed in Table 1. As seen from these values, the C atoms at positions 4 and 6 have the highest spin densities among the C atoms available for bonding. The heteroatom at position Y (or 1) also displays a relatively high spin density, with Te at this position having the highest spin density among all atoms. The C atom at position 4 has a higher spin density than that at position 6. The C atom at position 3 also exhibits significant spin density, although it is lower than those at positions 4 and 6.

**Table 1:** Mulliken Atomic Spin Densities of the Monomer Cationic Radicals

	1	2	3	4	5	6
SeSe	0.341	0.020	0.195	0.398	–0.078	0.336
SeTe	0.476	–0.023	0.189	0.335	–0.068	0.301
TeSe	0.336	0.032	0.184	0.395	–0.083	0.341
TeTe	0.468	–0.011	0.180	0.338	–0.076	0.306

Since bonding between two C atoms at the sixth position of two monomers does not allow chain propagation through the same positions, it can be said that the most probable bonding occurs between the C atoms at positions 4 and 6. As there is no qualitative difference depending on monomer change, the spin density map of the TeTe cationic radical, drawn by VMD based on grid data generated by Multiwfn 3.8, is given in Figure 2. It is evident that the unpaired electrons are dominantly distributed among the heteroatoms at position 1 and the C atoms at positions 3, 4, and 6.

**Figure 2:**
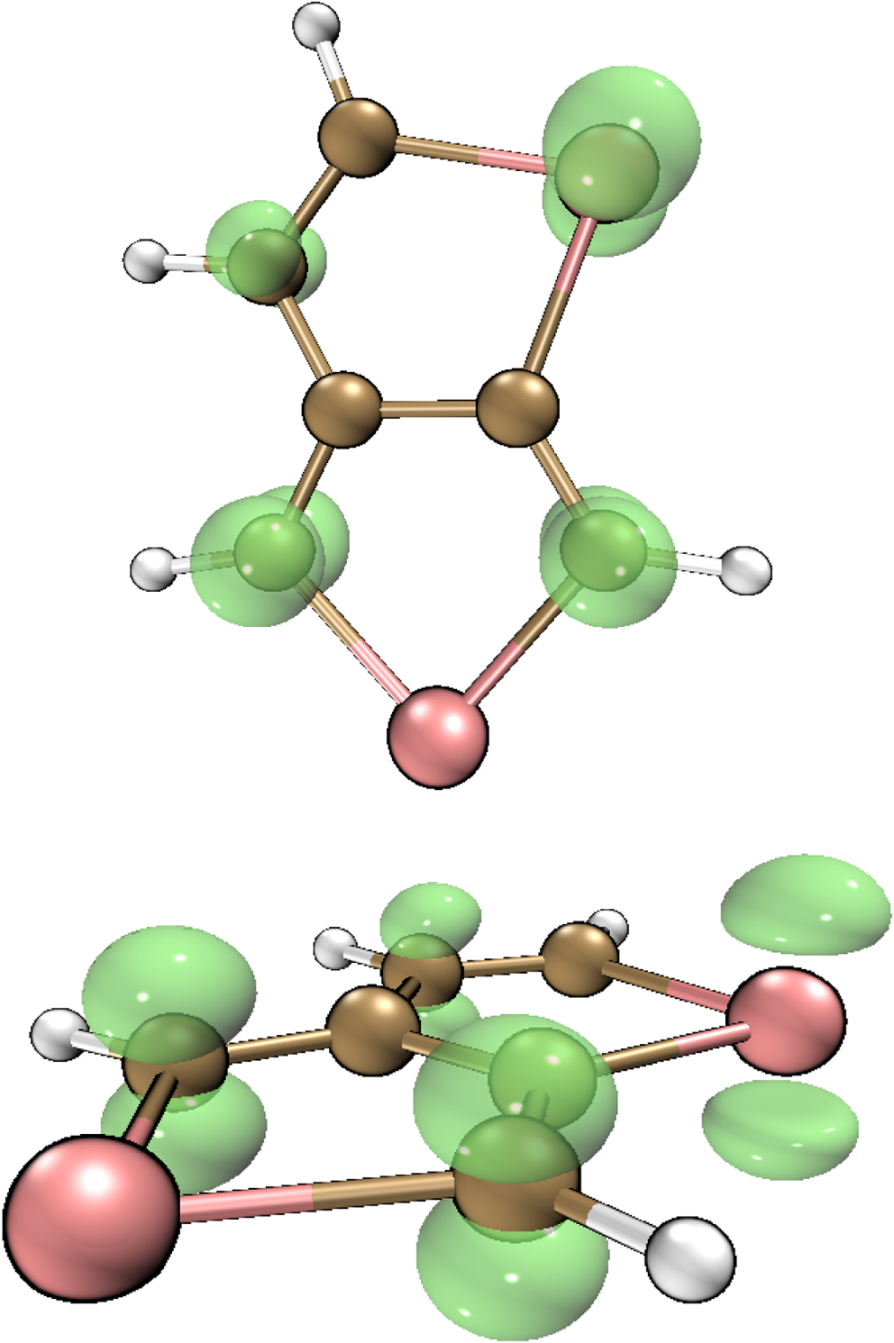
Spin density map of the TeTe cationic radical drawn by VMD based on grid data generated by Multiwfn 3.8 (from two different directions).

Another parameter used for quantitatively comparing potential reactive sites is the average local ionization energy (ALIE).^49^ Lower ALIE values indicate less tightly held electrons, suggesting that molecular regions with lower ALIE values are more favorable sites for reactions with electrophiles or radicals.^50^ There are four possible position pairs that are suitable for linear chain propagation: 2–4, 2–6, 3–6, and 4–6.

ALIE analysis on the molecular surface of the monomer was performed by using Multiwfn, and the surface maps were visualized by VMD, as shown in Figure 3. Cyan spheres on the map indicate the positions of ALIE minima on the surface. Consistently, three minima are found around the two heteroatom positions and somewhere between C atoms at positions 2 and 3. However, minima were not consistently observed around the carbon atoms at positions 4 and 6, which were identified as the most reactive sites based on spin density distribution. However, this does not imply the presence of tightly bound local electrons at these sites. The color transition is Blue-White-Red from the lowest ALIE values to the highest ALIE values. These sites have a blue color, which means that electrons are relatively easier to remove from these positions compared to other sites. Based on the combined analysis of spin density distribution and ALIE, positions 4 and 6 are identified as the most probable sites for oligomerization.

**Figure 3:**
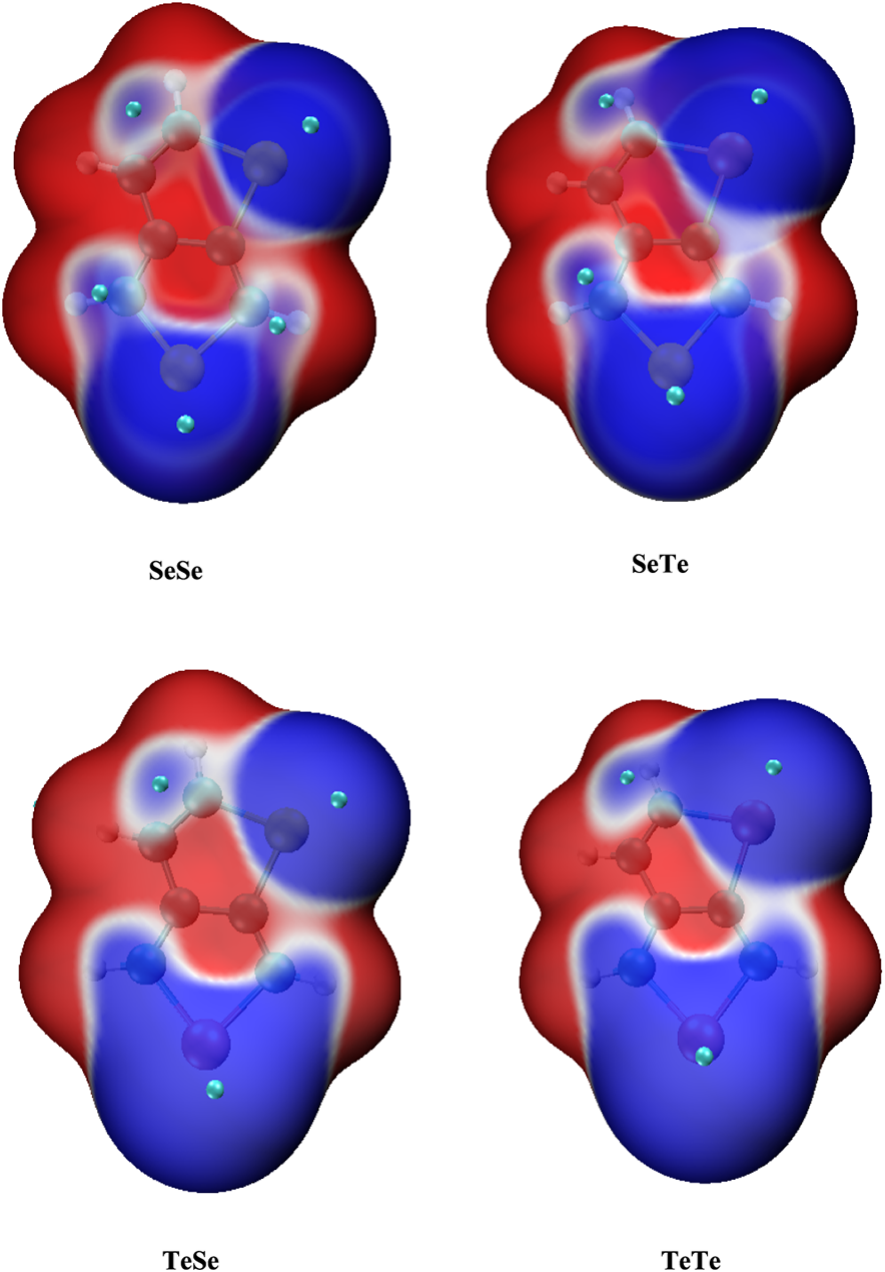
Molecular surface maps of average local ionization energy analysis (ALIE) on the molecular surface of the monomer (plotted by VMD).

### 3.2 Frontier Orbitals of the Monomers

Although some of the properties of the monomers change gradually upon chain propagation, their properties may provide insights into the prediction of the properties of polymer chains. The monomers examined in this study were previously investigated by Novak, who concluded that compounds with these specific heteroatomic positions exhibit the highest aromaticity, as determined by comparisons among structurally related molecules with different heteroatoms.^34^ In addition to the reactivity discussion of other monomer sites in the previous section, some aspects of these monomers are discussed here. The HOMO and LUMO shapes are given in Figures 4 and 5 (generated by using Avogadro). As seen, these orbitals have predominantly exhibited pi character, which is composed of p_*z*_ orbitals. However, the LUMOs of SeTe and TeTe have appreciable s contributions. A common property of these molecules is the presence of a Te atom at the Y position. Their atomic orbital compositions are given in Table 2, and the percentage of s orbital contributions to their LUMOs is 8.80% and 7.99%, respectively. As seen in Figures 4 and 5, the LUMO shapes of these molecules differ from the LUMO shapes of the other two molecules. All of the HOMOs and LUMOs of SeSe and TeSe have similar orbital shapes, primarily composed of atomic p_*z*_ orbitals. A detailed analysis of the frontier orbitals using the Multiwfn program revealed that these orbitals are composed of s, p_*x*_, and p_*y*_, while the others are predominantly composed of p_*z*_ orbitals (Table S1). Therefore, the substitution of Te at the Y position results in the disappearance of the pi character of LUMOs. In summary, planarization increases the pz orbital contribution, whereas PXTe structures exhibit an unusual s-orbital involvement. The orbital delocalization index (ODI), which indicates the degree of the extent of orbital spatial delocalization,^41^ is also given in the same table. Since lower ODI values indicate high spatial delocalization, the LUMOs of SeSe and TeSe have the greatest extension over the structure.

**Figure 4:**
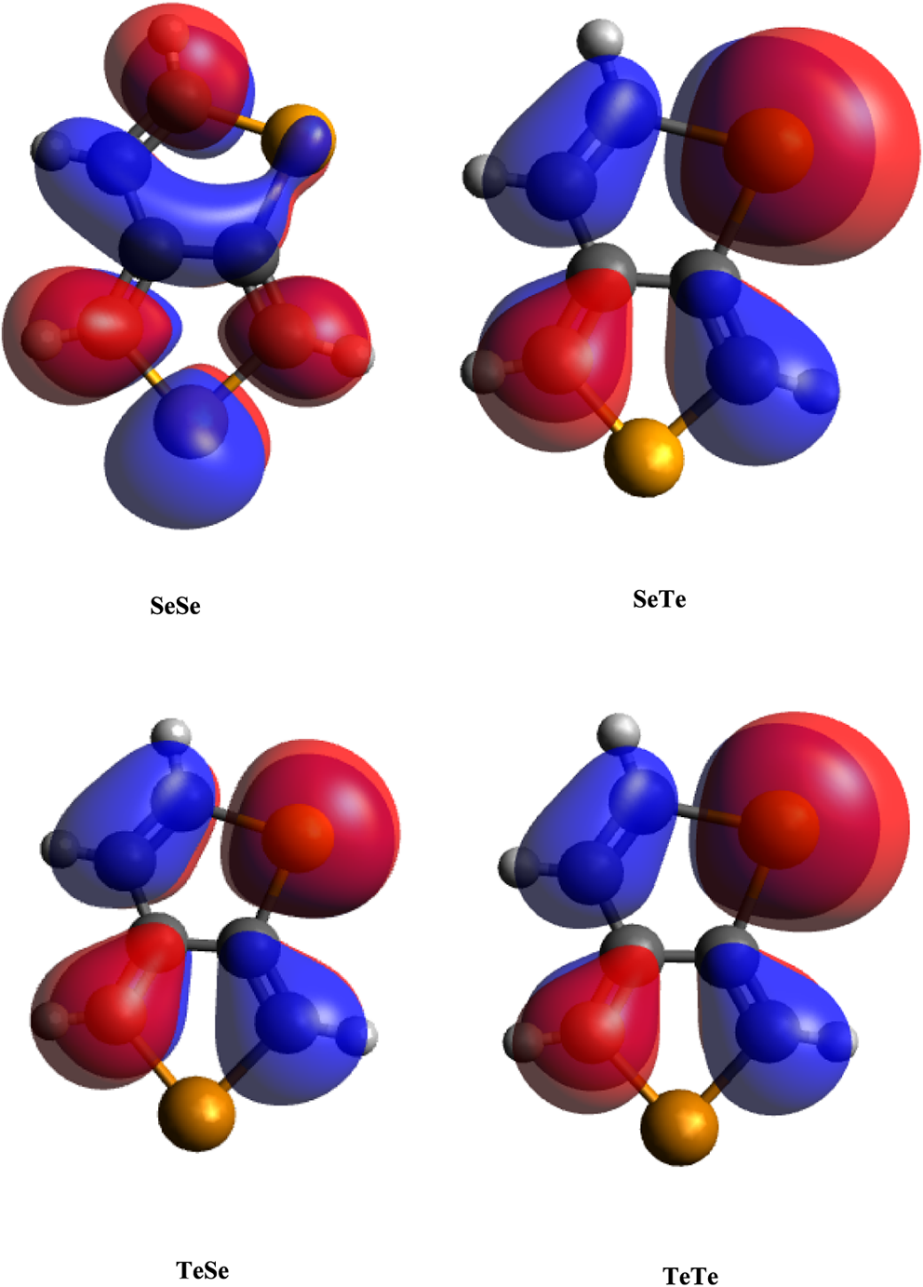
HOMOs of the monomers.

**Figure 5:**
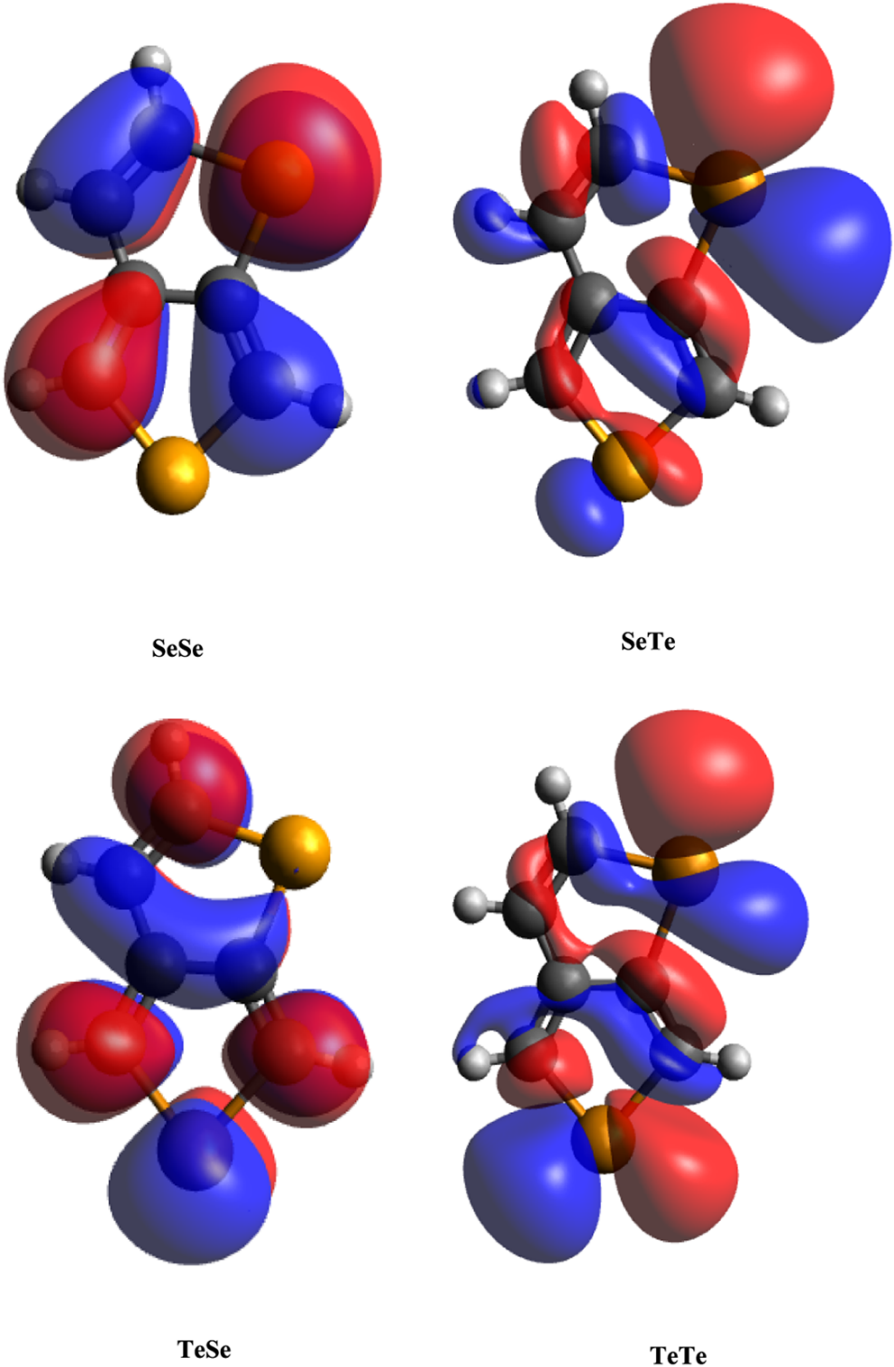
LUMOs of the monomers.

**Table 2:** Some Frontier Orbital Properties of the Monomers (Energy Values in eV)

Molecule		*E*	*E*_g_	s %	p %	d %	ODI	Atomic Compositions			
SeSe	HOMO	–5.62		0.00	99.21	0.80	23.95	11(Se)	0.09	12(Se)	37.13
SeSe	LUMO	–1.12	4.50	0.00	98.71	1.30	18.85	11(Se)	12.68	12(Se)	0.85
SeTe	HOMO	–5.43		0.00	99.37	0.63	31.99	5(Te)	50.52	12(Se)	0.00
SeTe	LUMO	–1.13	4.30	8.80	90.65	0.55	48.60	5(Te)	67.43	12(Se)	3.88
TeSe	HOMO	–5.52		0.00	99.21	0.79	23.95	11(Se)	37.52	12(Te)	0.18
TeSe	LUMO	–1.18	4.34	0.00	98.72	1.28	18.95	11(Se)	0.57	12(Te)	12.19
TeTe	HOMO	–5.34		0.00	99.37	0.63	32.15	5(Te)	0.01	6(Te)	50.85
TeTe	LUMO	–1.27	4.07	7.99	91.56	0.45	29.51	5(Te)	38.00	6(Te)	36.72

The HOMO and LUMO energy levels change gradually, with their gap decreasing as the period number of the heteroatom increases. Substitution of a Te atom into the Y position results in a greater increase in the HOMO level compared with substitution of the same atom into the X position. The contribution of the Te atom to the HOMO level is obviously higher for the SeTe monomer, and this trend also holds for comparable structures. In the TeSe structure, in which Se is in the Y position, Se is the heteroatom with the highest contribution to the HOMO energy level. When the structures with the same heteroatom in both positions are considered for HOMO energy levels, it can be generalized that the contribution from the Y position to the HOMO level is always greater than that from the X position. The HOMO energy level is correlated with the ionization energy, according to the Koopman theorem.^51^ The increase in the HOMO energy level due to the substitution of higher-period heteroatoms is consistent with the decrease in the ionization energy as the period number increases. A gradual decrease is also valid for LUMO energy levels. LUMOs are primarily contributed by the heteroatom in the X position for the structures with the same heteroatom in both positions, but this is only true for SeTe.

### 3.3 Relative Stabilities of the Polymers

Before the relative stabilities of the polymer structures are discussed, it should be noted that SeTe and TeSe are isomers of the same compound, formed by exchanging the positions of the two heteroatoms. The energy of SeTe is lower than that of TeSe by 1.117 kcal/mol. Therefore, it can be inferred that PSeTe isomers would have lower energy than PTeSe analogs. However, these two compounds are considered independently in order to compare their isomers among themselves. In Figure 6, the optimized geometries of PSeSe, with all the atoms restricted to the same plane, are shown with different connections.

**Figure 6:**
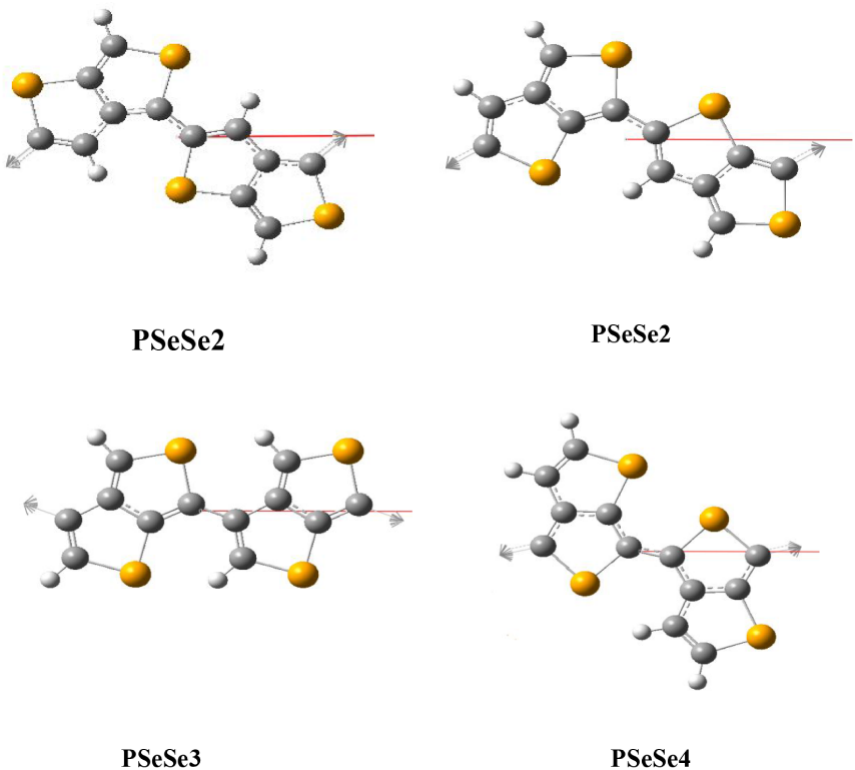
Optimized geometries of PSeSe (all of the atoms are restricted to the same plane).

As stated earlier, geometry optimizations were done without any assumptions other than that all of the atoms are in the same plane due to interchain interactions within the solid-state phase. The values of the dihedral angle, defined through the interring C–C bond with its neighboring C–C bond within the connected rings, for the optimized structures, along with the deviation energy (the energy difference between the planar structure and the structure that is obtained with nonrestricted optimization is given as *E*_planar_ – *E*_nr_ and called deviation energy) are shown in Figure 7. Since the dihedral angle is defined as 180° for planar geometry, the absolute value of the deviation of this angle from 180° is used as a measure of deviation from planarity. As seen in the figure, the highest deviation angles are obtained for the structures with 3–6 connections, except for PTeTe. The highest deviation occurs for the structures with a 3–6 connection, which arises due to the steric effect, which is most effective for this structure. All structures show some degree of deviation when no restriction for optimization is applied; however, the deviation angles for structures with 2–4 and 2–6 connections are very small compared to those of the others. For PTeTe structures, the highest deviation is obtained for the structure with 4–6 connection. However, the deviation energy for this structure is lower than that of the structure with a 3–6 connection, despite its higher deviation angle. This indicates that the steric effect on the structures with 3–6 connections is much stronger than that in the others. As a result, the deviation energy values for the structures with 3–6 connections are always the highest among them. Both the deviation angle and energy with connections 2–6 and 4–6 increase with the heteroatom Te substitution. The deviation energy versus the deviation angle is also plotted to see the correlation between them and shown in Figure S1. As seen, there is an exponential increase in the deviation energy with the deviation angle, although there are significant fluctuations due to the variations of the steric effect on molecular geometry. A question arises as to whether intermolecular interactions are strong enough to enforce planarity in these structures. While this lies beyond the scope of this study, it can be qualitatively stated that some structures with smaller deviations may achieve planar configurations (i.e., consecutive monomers are in the same plane), while others approach the planar configuration. In any case, the quantities obtained in this study belong to two frontier geometrical states. Theoretical values corresponding to their actual geometrical states should lie between them.

**Figure 7:**
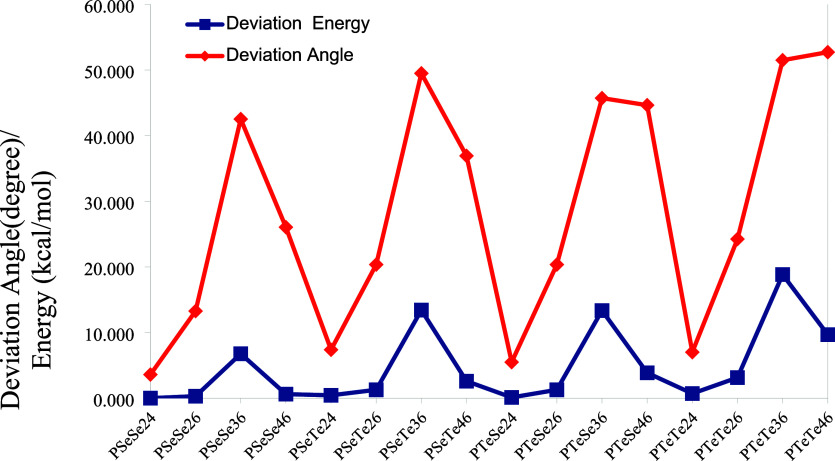
Deviation angles (from 180°) and deviation energies (*E*_planar_ – *E*_nr_) for each connection of polymer compounds.

The relative energies of the isomers, with respect to the smallest isomer for each polymer compound, have been calculated and are shown in Figure 8. Each polymer has four nonplanar and four planar structures, with the nonplanar structures having lower energy than the planar ones. The lowest energy is consistently obtained for the nonplanar structures with 4–6 connections, with the exception of PTeSe. In structures with 2–4 connections, the energy difference between planar and nonplanar forms is very small, so they are expected to be planar in their solid-state forms. These energy differences are also very small for PSeSe26 and PSeSe46. These findings indicate that structures with 3–6 connections make an insignificant contribution to the overall population due to their considerably higher relative energies.

**Figure 8:**
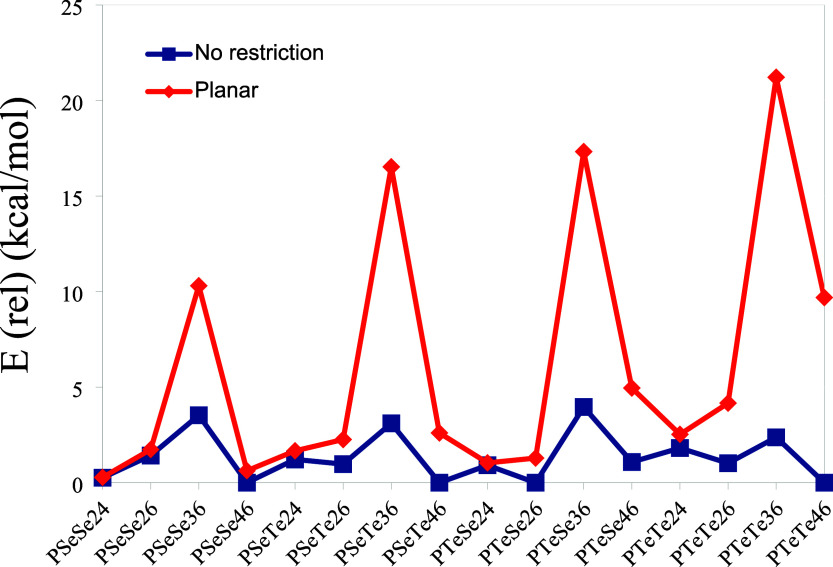
Relative energies of the isomers with respect to the isomer with the smallest energy for each polymer compound.

### 3.4 Frontier Orbitals of the Polymers

The HOCOs and LUCOs of PTeTe for their planar forms are shown in Figures 9 and 10. (The same orbitals for PSeSe are also shown in Figures S2 and S3). These orbitals generally show conjugated π orbital character, with their nodal planes fitting the monomer planes, with the exception of the LUCO of PTeTe26. The contributions of p_*x*_, p_*y*_, and p_*z*_ orbitals for this crystal orbital are 51.0%, 36.0%, and 0.0%, respectively. In contrast, its nonplanar form shows contributions of 26.2%, 37.0%, and 28.3% for the same orbitals (Table S1). Therefore, the p_*z*_ component is diminished as a result of the planarization. The LUCO of the nonplanar form of PTeTe36 has orbital contributions of 14.1%, 37.8%, and 38.5%, respectively, and its shape also deviates markedly from the conjugated π orbital appearance. On the other hand, all other orbitals have a p_*z*_ component exceeding 50.0%, and 42 out of 64 frontier orbitals of 32 structures have the p_*z*_ component higher than 90.0%. Among these, only 11 orbitals belong to nonplanar structures. This statistically shows that the planarization makes the p_*z*_ component more dominant. The percentage contributions of atomic s, p, and d orbitals for each polymer are given in Table S2. All of the frontier crystal orbitals are predominantly composed of p orbitals. While d-orbital contributions are around 1–2%, s-orbital contributions vary between 0.00% and 8.48%. The LUCOs of PTeTe26, PTeTe26p (where the suffix “p” denotes planar geometry), and PTeTe36 exhibit relatively high s-orbital contributions with contributions of 2.93%, 8.48%, and 2.46%, respectively. These crystal orbitals are also the orbitals mentioned above for the low p_*z*_ contribution. As can also be seen from their shapes, sigma orbitals are formed with remarkable s-orbital contributions.

**Figure 9:**
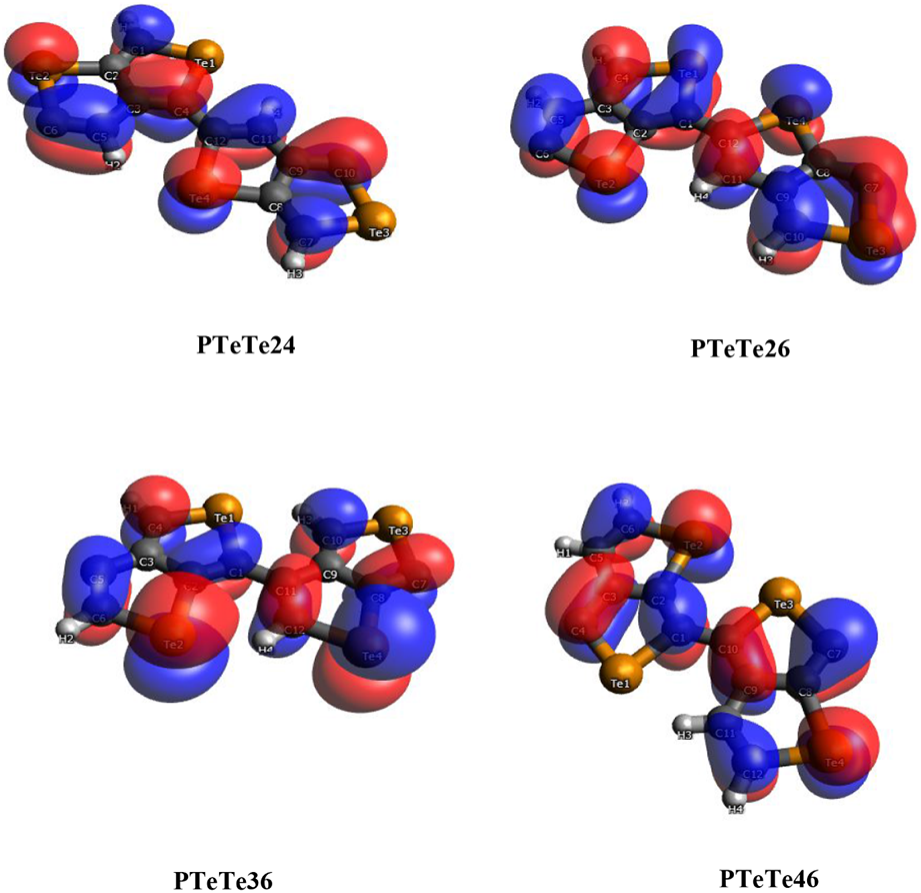
HOCOs of PTeTe for a planar geometry.

**Figure 10:**
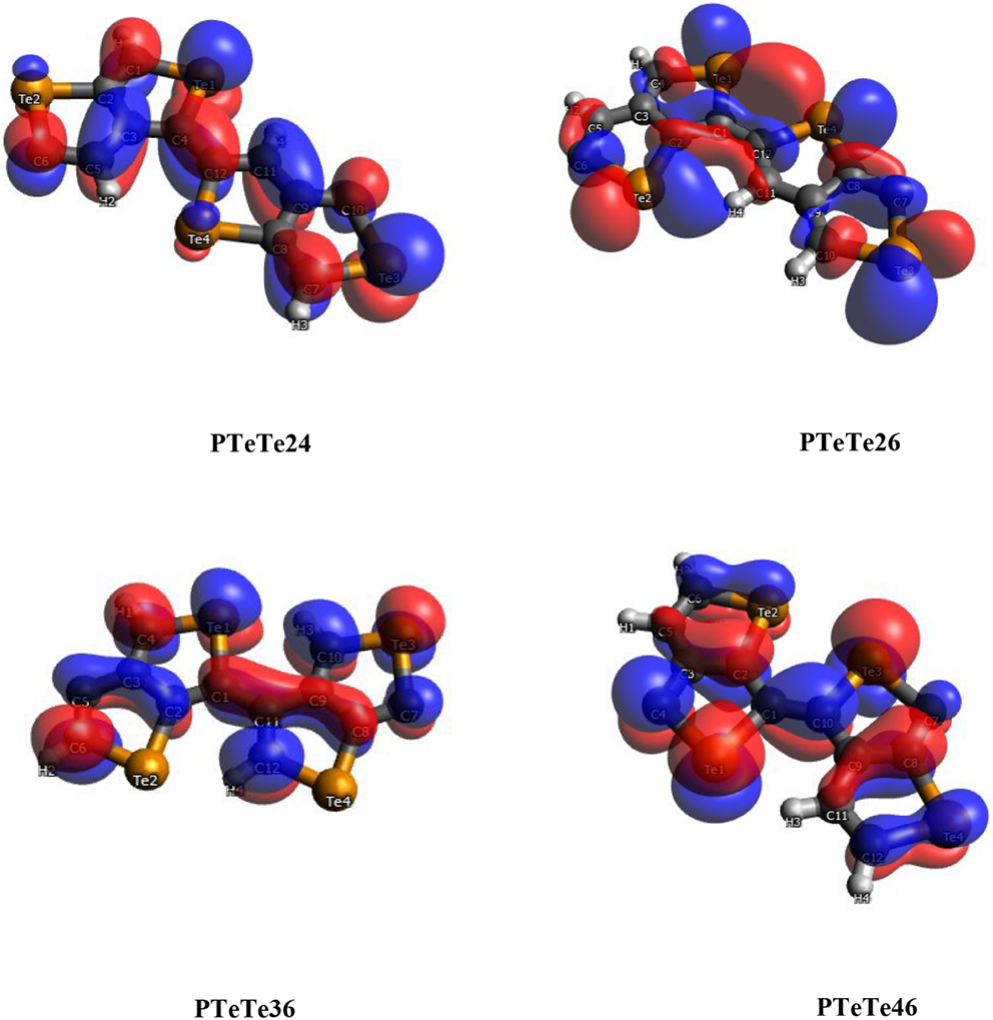
LUCOs of PTeTe for the planar geometry.

The orbital delocalization index (ODI) of the monomers was previously discussed. Their ODI values range from 18.85 to 48.60, with an average of 28.49. For the polymers, the range narrows to 7.56 and 14.03, with an average value of 10.07 (Table S2). However, it should be considered that each cell that is used to model a polymer chain comprises two monomers. Therefore, considering the ODI equation, approximately half of the monomer ODI values should be taken for comparison with the polymer.^41^ The ODI values corresponding to the frontier orbitals for the polymers are plotted in Figure 11. The ODI values of the planar structures are higher than those of nonplanar structures; i.e., the extension of the orbitals of nonplanar structures is greater than that in planar structures. The average ODI values of the HOCO/LUCO for nonplanar and planar forms are 10.07/9.11 and 10.86/10.23, respectively. These findings show that polymerization leads to an increased level of delocalization. It is worth noting that the ODI value approaches zero for an infinitely long polymer chain. However, structures with the same number of units must be considered to get a meaningful comparison of the orbital distribution due to their formulation. On average, LUCOs exhibit spatial delocalization greater than that of HOCOs. The lowest ODI values are observed for structures with 2–4 connections, while the highest values correspond to structures with 3–6 connections. The change in ODI of the frontier crystal orbitals upon planarization, defined as (ODI (planar) – ODI (nonplanar)), is plotted against the deviation angle (from 180°) in Figure 12. There is an approximate linear relationship between them, indicating that increased deviation from planarity correlates with lower spatial delocalization.

**Figure 11:**
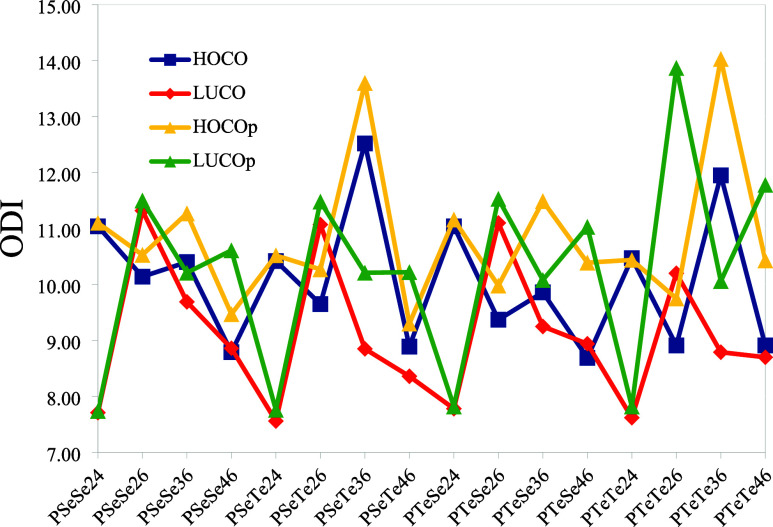
Orbital Delocalization Index (ODI) of frontier crystal orbitals (the suffix p indicates planar geometry).

**Figure 12:**
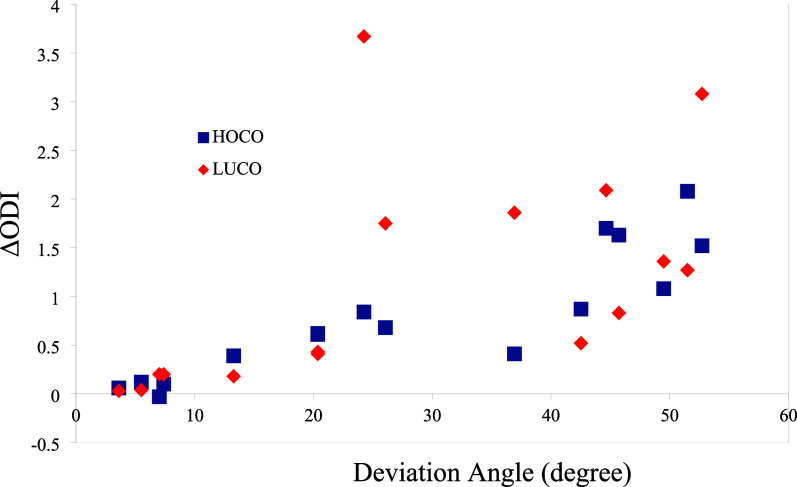
Change in the Orbital Delocalization Index (ODI) of frontier crystal orbitals versus the deviation angle (from 180°).

As is well-known, nearly continuous energy bands form in periodic structures due to the interaction of the molecular orbitals from neighboring repeating units. The energy level of the upper edge of the valence band (HOCO) shifts to a higher energy level, while the lower edge of the conduction band (LUCO) shifts to a lower energy level relative to the frontier orbital energy levels of the monomer due to polymerization. The absolute values of these shifts (monomers to planar polymers) are given in Figure 13. The shift in the LUCO energy level is greater than that of the HOCO level, indicating that the LUMOs of the neighboring monomers interact more strongly than the HOMOs. Therefore, the reduction in the energy gap is primarily contributed by the interactions of LUMOs. The highest shift in frontier orbital energy levels occurs in the structures with 4–6 connections, whereas the lowest is obtained for those with 3–6 connections. Thus, the largest interactions between frontier orbitals of neighboring units occur in the structures with 4–6 connections.

**Figure 13:**
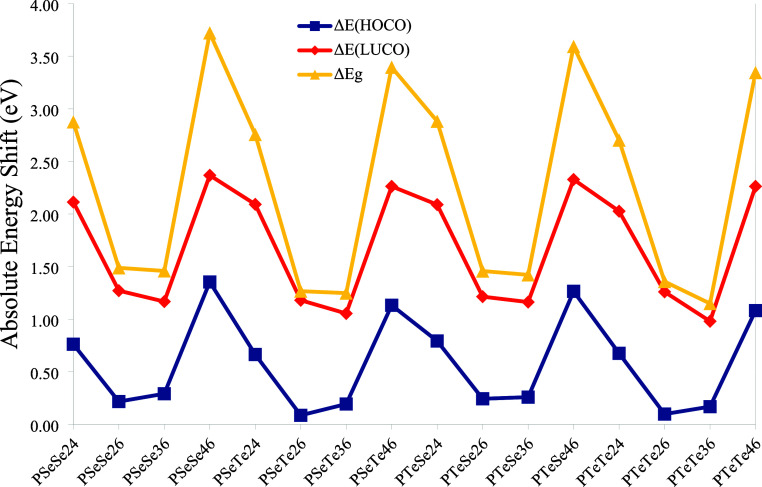
Absolute shift values (in eV) of the frontier orbital energy levels and band gaps from monomer to polymer (planar).

Figure 14 shows the HOCO and LUCO energy levels, along with the corresponding band gap values (in eV). As expected, increased planarity of geometry leads to a reduction in band gap values. The delocalization of the π electrons increases due to the planarity of the aromatic backbone, and this causes a reduction in the gap between HOCO and LUCO.^52^ The extent of this reduction strongly depends on the deviation angles, which are given in Figure 7. To further analyze the relationship, the reduction in the band gap as a function of deviation angle is plotted and shown in Figure 15 The results suggest an approximate linear correlation between the band gap reduction and deviation angle, accompanied by significant fluctuations. These high fluctuations likely arise from the atomic orbital interactions, which are dependent on the geometry resulting from connection sites, heteroatoms, and the positions of heteroatoms. It is noticeable that the grouping of PXY36 and PXY46 occurs within the same deviation angle region. PXY46 exhibits greater sensitivity to changes in deviation angle, and its reduction increases with deviation angle. However, there is a very small change, even a small decrease, in the deviation angle for PXY36. The final two points for PXY36 belong to the polymers PSeTe36 and PTeTe36, respectively. Overall, the results demonstrate that the degree of reduction is determined by multiple structural factors, including the deviation angle, the nature of heteroatoms, connection sites, and positions of heteroatoms. The HOCO energy levels of PXY46 exhibit a significant increase when the structure transforms from nonplanar to planar. However, no noticeable changes are observed in the structures with different connection sites. There is a significant decrease in the LUCO levels of PXY36 and PXY46 in general. It must be added that there has been a remarkable decrease in the LUCO level as well as in PTeTe26. As a result, the band gap reduction in PXY46 arises from shifts in the energy levels of both frontier orbitals, as the LUCO energy level shift is responsible for the PXY36 band gap decrease.

**Figure 14:**
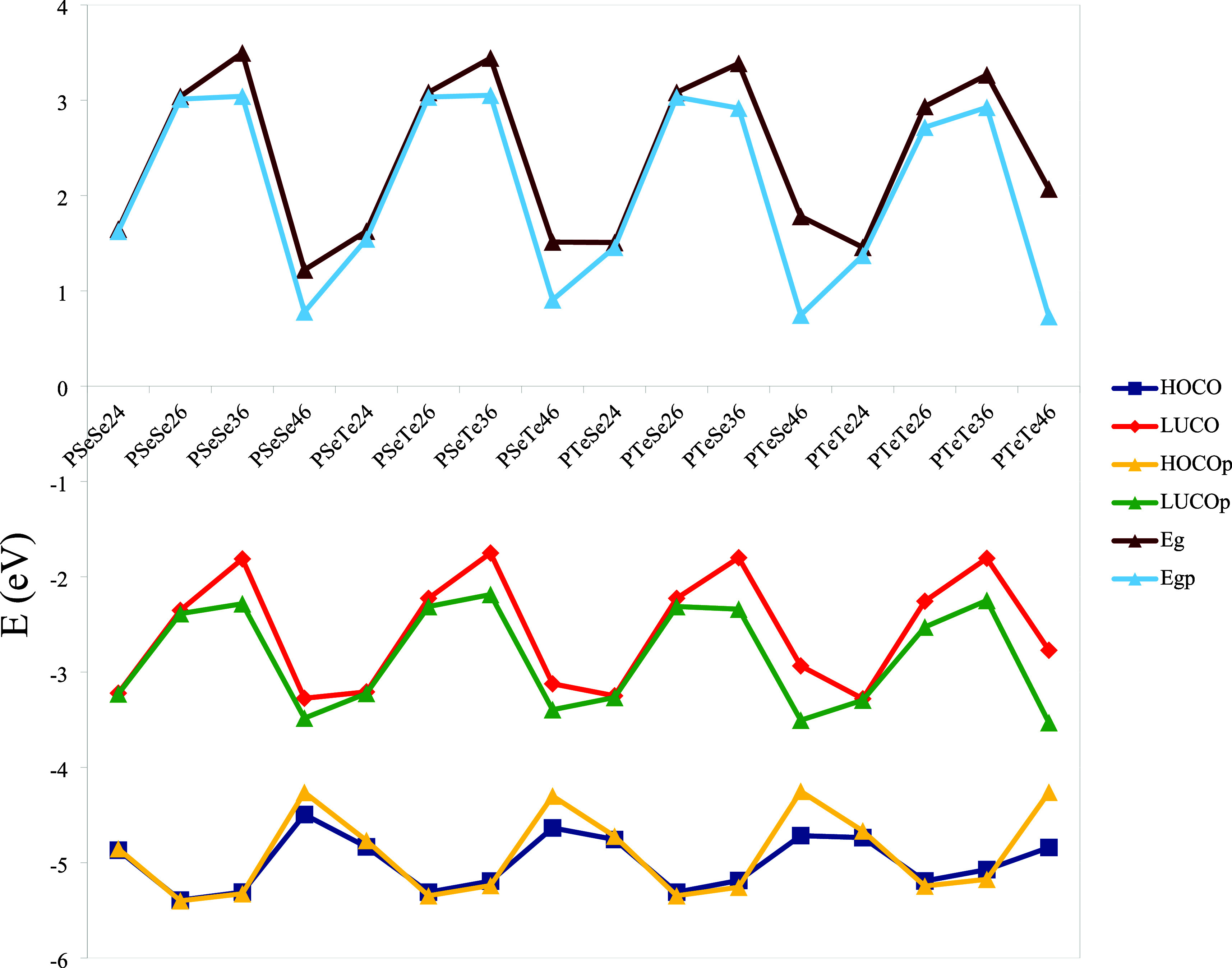
HOCO and LUCO energy levels and band gap values (the suffix p indicates planar geometry).

**Figure 15:**
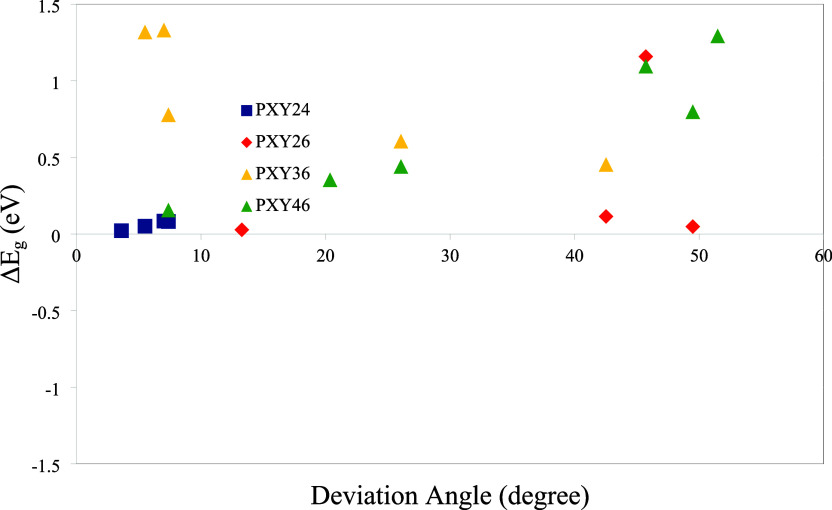
Reduction of the band gap as a function of the deviation angle.

The band gap value of PSeSe was determined experimentally to be 0.76 eV by Patra et al.^15^ In the same study, the band gap of the same polymer with 4–6 connections was calculated to be 0.83 eV by using the B3LYP/6-31G­(d) method. Our calculated band gap value for this structure is 0.78 eV, which is slightly closer to the experimental value than that reported by Patra et al.^15^ In the same study, the band gaps of PSS, PSSe, and PSeS were measured to be 0.85, 0.96, and 0.72 eV, respectively, while the calculated values for 4–6 connections were 0.96, 1.07, and 0.69 eV for the same polymers. It is interesting to obtain higher band values when Se was substituted into the Y position instead of S. On the other hand, PSeS, which has the same skeleton as PSS, has a lower band gap value compared to PSS. A similar trend was observed for the PSeSe, PSeTe, and PTeSe in our results, with band gap values of 0.78, 0.91, and 0.75 eV, respectively (Table S2). The variation of band gap as a function of heteroatom position has been studied by several authors.^53–55^ Hutchison et al. proposed that the electron affinity of the heteroatom influences the band gap.^4^ Considering the LUCO values (Table S2), the contribution of the Te atom at the X position is higher than that at the Y position. This may account for the lower band gap for PTeSe compared to PSeSe and PSeTe. We have performed additional calculations for the 4–6 connections to explore the heteroatom effect by changing the basis set to SDD, which also covers Po.^40^ The results are listed in Table S3. In this table, the elements given in each column represent the element at the X position, and the element that appears in each row represents the element at the Y position. The same effect is observed for PTeTe, PTePo, and PPoTe, with the band gaps of 0.717, 0.764, and 0.686 eV, respectively. The lowest band gap was obtained for PPoPo, as expected, with a value of 0.686 eV. The results clearly indicate that different heteroatom combinations give interesting systematic properties, which may be the subject of a future study involving a detailed analysis of all group elements.

Because molecular orbitals are composed of atomic orbitals, orbital and atomic compositions of frontier orbitals were calculated and are listed in Tables S1 and S2. Each molecular orbital is formed by the combinations of C atoms and heteroatoms located at different positions with different coefficients, resulting in different shapes and different energy levels. Since all atoms other than heteroatoms are the same, the heteroatoms are primarily responsible for modifying molecular orbital properties. Each heteroatom, depending on its chemical identity and position, affects frontier orbital energy levels. Electronegativity and electron affinity,^4^ which are related to atomic orbitals, and polarizability^20^ are the important parameters that have previously been identified as important parameters correlated to band gap. Band gap changes also have been associated with the aromaticity of cycles, including that heteroatom. In the study by Patra et al.,^15^ it was shown that the lower aromaticity in the main ring (the ring including the X position in the 4–6 connection) reduces the band gap due to the contribution of the quinoid structure of the main chain. But lower aromaticity in the peripheral ring (the ring including the Y position in the 4–6 connection) enhances the aromaticity of the main ring, resulting in an increase in the band gap due to a decrease in the quinoid character of the main chain.^15^ Compared to other chalcogenophenes (the aromaticity order: thiophene > selenophene > tellurophene > furan), tellurophene has a relatively lower aromaticity.^30^ Therefore, the band gaps of all the polymers can be discussed in terms of the relative aromaticity of the fused rings. Since enhancing the quinoid character of the polymer backbone leads to lower band gap polymers,^15^ the lower aromatic character of the ring covering the polymer backbone part causes a lower band gap. The same rationale can be used for the PSeSe, PSeTe, and PTeSe for the 4–6 connection. Since the selenophene ring is more aromatic than tellurophene, the tellurophene ring makes selenophene more aromatic. This results in a higher band gap value (0.91 eV) than that of PSeSe (0.78 eV). The same explanation applies to the PTeTe, PTePo, and PPoTe polymers.

### 3.5 Band Structures

The energy of the charge carriers (electrons in the conduction band and positive holes in the valence band) as a function of the wave vector is called the band structure. Several important parameters relevant to material applications can be derived from the band structure, including the HOCO and LUCO energy levels, band gap, bandwidth, and effective mass. The first three parameters were discussed in the previous section. In this section, the bandwidth and effective mass for both the valence and conduction bands are analyzed in the context of intrinsic conductivity.

The length of a one-dimensional cell is defined as translational vectors (Tv) due to translational symmetry within the periodic boundary conditions.^36^ The translational vector lengths of the polymers are given in Figure 16. These lengths vary depending on the connection positions within the same monomeric unit. The shortest lengths are observed for structures with 4–6 connections, while the longest lengths occur for structures with 2–6 connections. It is evident that the orientation of the two monomers within the cell also determines the cell size (Figure 6). Planarity has a significant effect only on the structures with 3–6 connections, as the cell dimensions are enlarged due to the restriction of keeping all the atoms on the same plane.

**Figure 16:**
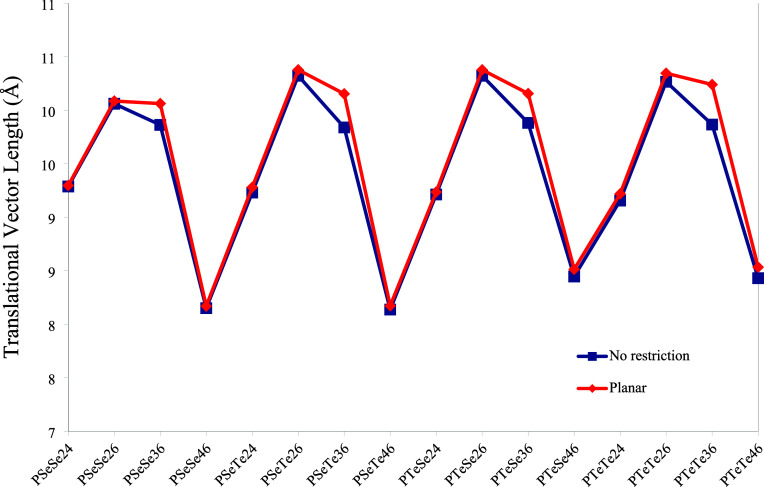
Translational vector lengths of the polymers.

The band structures of planar PTeSe in the first Brillouin zone as a function of different connection positions are given in Figure 17. The band structures have a similar appearance but differ in numerical parameters. Therefore, the band structures of other polymers are not provided. In the figure, five occupied and five unoccupied band states are depicted. The two curves in the middle show each polymer’s valence band and conduction band. As seen, the minimum energy difference between the valence and conduction bands always occurs at the same k-point, the gamma (Γ) point. Therefore, they are all direct band gap polymers.

**Figure 17:**
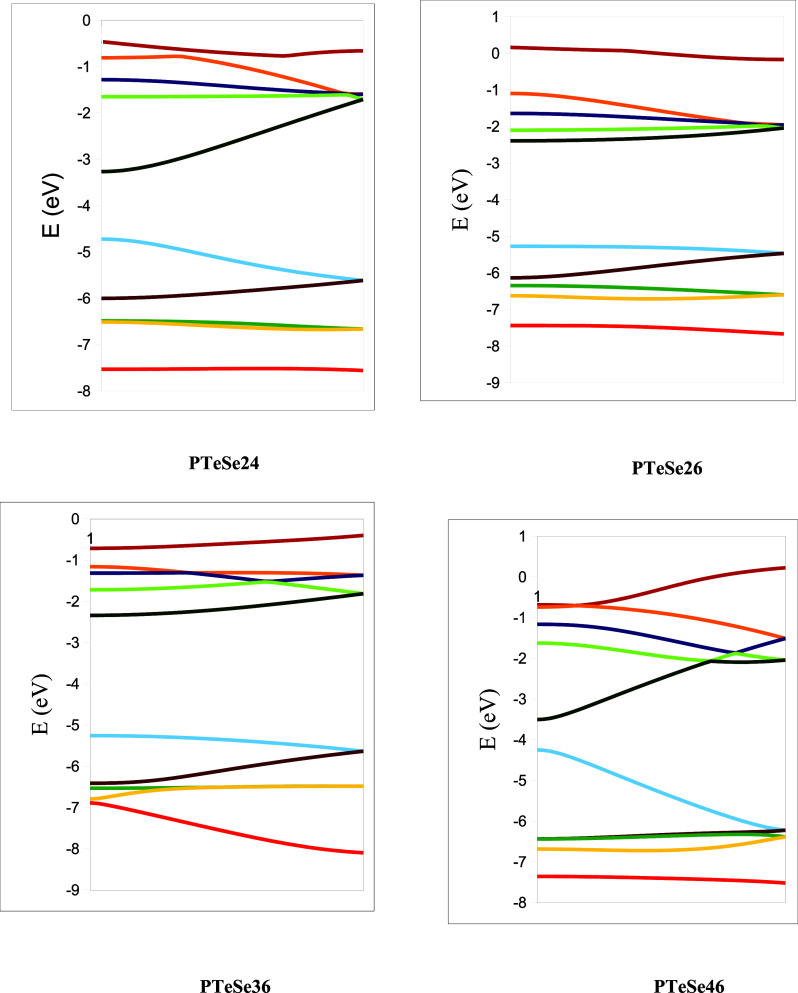
Band structure of planar PTeSe.

As is well-known, bands are formed due to the overlapping of orbitals along the chain, and the bandwidths increase with the degree of overlapping of the corresponding orbitals.^56^ The bandwidths of the polymers are depicted in Figure 18. The highest bandwidths are obtained for planar structures with 4–6 connections, while the polymers with 2–4 connections also have bandwidths comparable to those with 4–6 connections. The high bandwidths of the planar structures arise from the fact that planarity brings their atoms closer together. This interpretation also applies to polymers with 4–6 and 2–4 connections. The lowest translational vectors are also obtained for the polymers with 4–6 connections. As is known, low bandwidth indicates the localization of charge carriers, which in turn reduces the mobility of charge carriers.^57^

**Figure 18:**
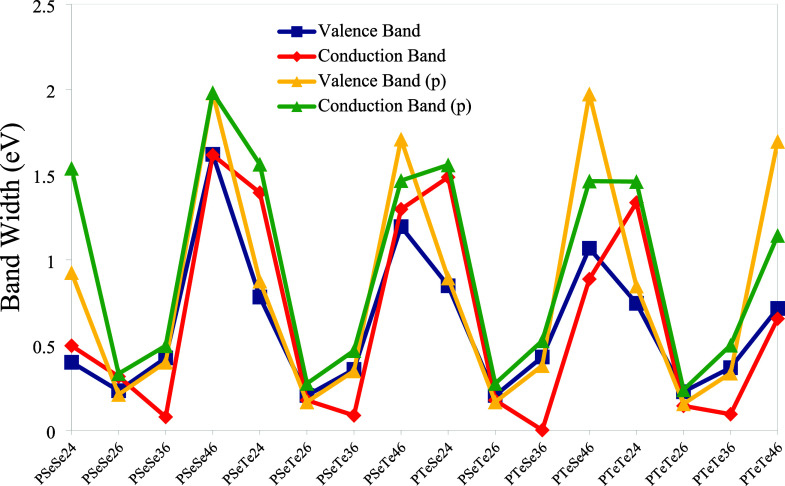
Bandwidths of the polymers (p indicates planar structure).

Another parameter related to the band structure is the effective mass. In periodic structures such as polymers, charge carriers, positive holes in the valence band, and electrons in the conduction band can be described as free electrons with an effective mass. The effective mass can be calculated using the curvature of the band structure near the band gap.^58^ To determine the effective mass, several points near the Γ point (Figure 17) were used to fit a second-order polynomial function. The effective mass formula^58^ is then used to calculate effective mass values, which are given in Figure 19. The lowest effective mass values are obtained for the polymers with 4–6 and 2–4 connections. It should be noted that since the valence band of PTeSe36 diverges to infinity, it is not shown in the figure. Within a certain limit, there is inverse proportionality between the effective mass and the energy bandwidth.^58^ Our results also show this relationship approximately (Figure 20).

**Figure 19:**
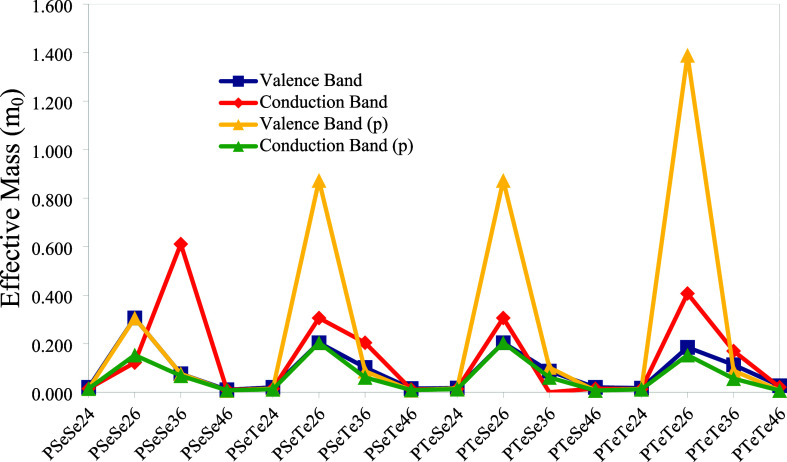
Absolute values of the effective mass of electrons in the valence band and positive holes in the conduction band (in the unit of rest electron mass (*m*_0_)).

**Figure 20:**
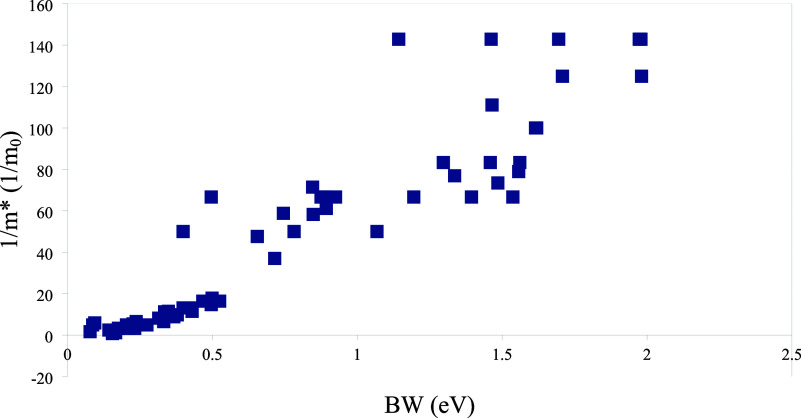
Reciprocal of effective mass (1/*m*_e_) versus bandwidth (eV).

The band structures of polyselenophene (PSe), along with polypyrrole (PPy) and polythiophene (PTh), which are commonly studied and used for several applications, were calculated by B3LYP/6-31G­(d).^46^ The B3LYP gives band structure values comparable to those obtained using B3PW91, indicating consistency with our results. In this study, the band gap values of PPy, PTh, and PSe are greater than those of the polymers with the 4–6 connection. As the band gap value decreases, the population of electrons in the conduction band and positive holes in the valence band increases for the intrinsic conductivity. Therefore, the polymers investigated in this study have the advantage of higher charge carrier populations than those of PPy, PTh, and PSe. The calculated valence and conduction bandwidth values for PPy, PTh, and PSe are 2.239/1.488, 2.160/1.870, and 1.976/1.957 eV, respectively. As seen from Figure 18, the valence bands of PSeSe46 and PTeSe46, in particular, have bandwidth values comparable to those of PPy, PTh, and PSe. The conduction band of PSeSe46 has a bandwidth similar to those of PTh and PSe. Since a reduced bandwidth indicates lower charge carrier mobility,^57^ the other bands of structures with 4–6 and 2–4 connections may exhibit slightly lower mobility. The calculated valence and conduction effective mass values for PPy, PTh, and PSe are −0.020/0.020, −0.015/0.015, and −0.015/0.015, respectively. The absolute value of effective mass for valence and conduction bands of the title planar polymers with 4–6 and 2–4 connections is around 0.008 and 0.015 *m*_0_, respectively. Although there is no direct relationship between effective mass and mobility due to factors such as electronic structure, scattering mechanisms, and anisotropy,^59^ effective mass has a negative contribution to mobility. Therefore, the planar structures of the molecules studied have an advantage over PPy, PTh, and PSe for the contribution of the effective mass to mobility.

Semiconducting polymers can be used for various organic electronic devices.^2,60–62^ The polymers examined in this study can be used for numerous applications depending on their stability under certain conditions. One of the key parameters for these applications is the band gap. The band gaps of the title polymers of this study range from 0.73 to 3.53 eV, classifying them as semiconductors.^60^ According to basic solar cell theory, the optimum band gap is approximately 1.34 eV.^2^ Most of the polymers studied here with 2–4 connections have band gap values close to this value, within the expected calculation error range. Polymers with band gaps lower than 1.5–2.0 eV are classified as low band gap, and they are suitable for optoelectronic applications.^2^ The polymers studied here with 2–4 and 4–6 connections fall within this region, indicating their potential use in such applications. Specifically, these polymers fit in the near-infrared (NIR) region (0.4–1.59 eV), and therefore, these polymers can be used for harvesting photons in the NIR region of the solar spectrum. They may be suitable for the fabrication of near-infrared photodetectors and near-infrared light-emitting diodes (biosensors, security applications, etc.).^2^

## 4 Conclusions

In this study, polymers composed of bicyclic fused cycles with two heteroatom positions were analyzed by employing a hybrid functional within the periodic boundary conditions. The heteroatoms Se and Te are alternatively considered for these two positions. As a result of the analysis of monomer cations, based on the electrochemical polymerization assumptions, positions 4 and 6 are particularly found to be more reactive positions to make bonding during oligomerization. This conclusion is supported by the spin density distribution and ALIE analyses. The LUMOs of SeTe and TeTe have significant s-orbital contributions of 8.80% and 7.99%, respectively, indicating that the substitution of Te at the Y position results in s-orbital contribution to the LUMO. In general, replacing Se with Te in the monomer results in an increase in the HOMO energy level and a decrease in the LUMO energy level, resulting in a reduction in the HOMO–LUMO energy gap. The aromatic character of the heterocycles explains the increase in the band gap upon Te substitution at the Y position in PSeTe. Based on the results, polymers with lower band gaps can be anticipated, considering that the method used in this study yields deviations of approximately 10% from experimental values.^38^

Polymer geometries, defined using a cell containing two monomers with different connections, were optimized by considering both planar and nonplanar forms. Planar forms were found to have higher energies compared to those of their nonplanar forms. In the case of a relatively small deviation from planarity with small energy differences, planar structures are expected due to interchain interactions in the solid state. In other cases, at least the geometry gets closer to the planar form. Therefore, the properties of planar forms with small energy deviation can serve as reliable predictors, while those with significant deviations can be estimated using geometries intermediate between planar and nonplanar forms.

The relative stabilities of isomers were analyzed by comparing their energies, and the smallest energy is always obtained for the nonplanar structures with 4–6 connections, with the exception of PTeSe. On the other hand, structures with 2–4 connections, such as PSeSe26 and PSeSe46, have very small energy differences between their planar and nonplanar forms, so they are expected to be planar in their solid-state form. The structures with 3–6 connections have very high relative energies.

The orbitals of the polymers with planar structures generally show conjugated π orbital characters, with their nodal planes fitting the monomer plane, except for the LUCO of PTeTe26. The LUCOs of the nonplanar forms of PTeTe26 and PTeTe36 do not have dominant π orbital character. However, all other orbitals have a p_*z*_ component higher than 50.0%, indicating dominant π orbital character. The orbital delocalization index (ODI) increases upon polymerization. On the other hand, LUCOs, on average, have higher spatial delocalization than HOCOs. Moreover, spatial delocalization increases with the deviation from planarity,

As is known, intrinsic conductivity mainly depends on the band gap and mobility. As the band gap decreases, the population of positive holes in the valence band and electrons in the conduction band increases at a given temperature. Based on band gap, the title polymers with 2–4 and 4–6 connections are superior to the commonly studied semiconducting polymers polypyrrole and polythiophene, which have band gap values of 2.85 and 2.0 eV,^63^ respectively. Bandwidth and effective mass are correlated with each other, and an approximate inverse relationship between them is shown using the data produced in this study as well. As the bandwidth increases, mobility also increases, assuming that other parameters are negligible. The valence bands of PSeSe46 and PTeSe46 exhibit bandwidth values comparable to those of PPy, PTh, and PSe. The conduction band of PSeSe46 has a bandwidth similar to that of PTh and PSe. Therefore, the conductivities arising from these bands might have levels similar to those of these common polymers, disregarding other parameters. Other band structures with 4–6 and 2–4 connections also have noticeable bandwidths, considering PPy and PTh. Moreover, the values of effective mass, which negatively contribute to mobility, may be compared, although there is no direct relationship between effective mass and mobility due to factors of electronic structure, scattering mechanisms, and anisotropy.^59^ The absolute values of the effective mass of valence and conduction bands for the title planar polymers with connections 4–6 and 2–4 are approximately 0.008 and 0.015 *m*_0_, respectively. These values are smaller than or roughly equal to the calculated valence/conduction effective mass values of PPy, PTh, and PSe, which are −0.020/0.020, −0.015/0.015, and −0.015/0.015, respectively. Thus, in the context of effective mass, the title polymers with 4–6 connections demonstrate better conductivity than PPy, PTh, and PSe. Overall, the findings indicate that the planar structures of the title molecules offer a substantial improvement over PPy, PTh, and PSe regarding the contribution of effective mass to mobility. As a result, the title polymers with 4–6 and 2–4 connections may serve as promising candidates as semiconducting materials. Their values of deviation energy from planarity are less than 5 kcal/mol, except for PTeTe46, which has a deviation energy of approximately. Therefore, their solid forms are likely to be planar or nearly planar due to interchain interactions. Polymers with 2–4 and 4–6 connections specifically fall in the region of the near-infrared (NIR) 0.4 eV–1.59 eV. Therefore, they have potential applications in harvesting photons in the NIR region of the solar spectrum and in the fabrication of near-infrared photodetectors and near-infrared light-emitting diodes (biosensors, security applications, etc.).^2^

## Supplementary Material



## Notes

The sum of percent contributions of atomic p orbitals for the frontier orbitals of the monomers and polymers (Table S1); some properties of frontier orbitals of the polymers (Table S2); the band gap, HOCO, and LUCO values (in eV) calculated by B3PW91/SDD method (Table S3); the deviation energies (*E*_planar_ – *E*_nr_) versus the deviation angle (from 180°) (Figure S1); HOCOs of PSeSe for planar geometry (Figure S2); LUCOs of PSeSe for planar geometry (Figure S3) (PDF)
