# Supplementary material for: Low Band Gap Fused Bicyclic Polymers with Heteroatoms Se and Te: A DFT-PBC Study
Source: ACS Omega. 2026 Mar 2;11(10):15694–709. doi: 10.1021/acsomega.5c04257 (PMC13000587; doi:10.1021/acsomega.5c04257)
Supplement: Supplementary file 1 [file ao5c04257_si_001.pdf]

# Supporting Information

## Low Band Gap Fused Bicyclic Polymers with Heteroatoms Se and Te: A DFT-PBC Study

**Zeki Büyükmumcu[1*] and Fatma Selampinar[2]**

[1]*Department of Chemistry, Faculty of Sciences, Erciyes University, Kayseri 38039, Turkey*

[2]*Department of Chemistry, University of Connecticut, Storrs, CT 06269-3060, United States*

***To whom correspondence should be addressed: zekib@erciyes.edu.tr (Z.B.)**
**Fatma.selampinar@uconn.edu (F.S.)**

**Contents**


**Table S1.** The sum of percent contributions of atomic p orbitals for the frontier orbitals of the monomers and polymers. S3

**Table S2.** Some properties of frontier orbitals of the polymers. S4

**Table S3.** The band gap, HOCO and LUCO values (in eV) calculated by B3PW91/SDD method. S6

**Figure S1.** The deviation energies ($E_{planar}$-$E_{nr}$) versus the deviation angle (from 180°). S7

**Figure S2.** HOCOs of PSeSe for planar geometry. S8

**Figure S3.** LUCOs of PSeSe for planar geometry. S9

**Table S1.** The sum of percent contributions of atomic p orbitals for the frontier orbitals of the monomers and polymers (The sum of atomic p orbitals with threshold of absolute value greater than 0.5 %, the suffix p indicates planar form.)

| Polymer | Orbital | $p_x$ % | $p_y$ % | $p_z$ % | Polymer | Orbital | $p_x$ % | $p_y$ % | $p_z$ % |
|---|---|---|---|---|---|---|---|---|---|
| SeSe | HOMO | 0.0 | 0.0 | 98.3 | PSeTe46 | HOCO | 1.5 | 11.4 | 80.8 |
| SeSe | LUMO | 0.0 | 0.0 | 97.4 | PSeTe46 | LUCO | 0.0 | 14.4 | 76.4 |
| SeTe | HOMO | 0.0 | 0.0 | 99.0 | PSeTe46p | HOCO | 0.0 | 0.0 | 97.3 |
| SeTe | LUMO | 51.3 | 38.5 | 0.0 | PSeTe46p | LUCO | 0.0 | 0.0 | 96.6 |
| TeSe | HOMO | 0.0 | 0.0 | 98.2 | PTeSe24 | HOCO | 0.0 | 0.0 | 95.1 |
| TeSe | LUMO | 0.0 | 0.0 | 97.7 | PTeSe24 | LUCO | 0.0 | 0.0 | 95.0 |
| TeTe | HOMO | 0.0 | 0.0 | 98.9 | PTeSe24p | HOCO | 0.0 | 0.0 | 97.1 |
| TeTe | LUMO | 43.7 | 46.9 | 0.0 | PTeSe24p | LUCO | 0.0 | 0.0 | 98.0 |
| PSeSe24 | HOCO | 0.0 | 0.0 | 96.4 | PTeSe26 | HOCO | 0.0 | 3.4 | 89.2 |
| PSeSe24 | LUCO | 0.0 | 0.0 | 96.7 | PTeSe26 | LUCO | 0.0 | 6.8 | 86.8 |
| PSeSe24p | HOCO | 0.0 | 0.0 | 97.1 | PTeSe26p | HOCO | 0.0 | 0.0 | 98.1 |
| PSeSe24p | LUCO | 0.0 | 0.0 | 97.8 | PTeSe26p | LUCO | 0.0 | 0.0 | 98.0 |
| PSeSe26 | HOCO | 0.0 | 0.5 | 93.1 | PTeSe36 | HOCO | 0.0 | 26.3 | 67.1 |
| PSeSe26 | LUCO | 0.0 | 1.2 | 93.6 | PTeSe36 | LUCO | 4.2 | 27.0 | 56.8 |
| PSeSe26p | HOCO | 0.0 | 0.0 | 98.1 | PTeSe36p | HOCO | 0.0 | 0.0 | 98.6 |
| PSeSe26p | LUCO | 0.0 | 0.0 | 98.0 | PTeSe36p | LUCO | 0.0 | 0.0 | 97.3 |
| PSeSe36 | HOCO | 0.0 | 23.3 | 69.2 | PTeSe46 | HOCO | 4.8 | 11.9 | 75.3 |
| PSeSe36 | LUCO | 0.0 | 21.0 | 68.4 | PTeSe46 | LUCO | 0.0 | 19.9 | 73.4 |
| PSeSe36p | HOCO | 0.0 | 0.0 | 98.5 | PTeSe46p | HOCO | 0.0 | 0.0 | 97.7 |
| PSeSe36p | LUCO | 0.0 | 0.0 | 97.3 | PTeSe46p | LUCO | 0.0 | 0.0 | 97.4 |
| PSeSe46 | HOCO | 0.0 | 7.7 | 86.4 | PTeTe24 | HOCO | 0.0 | 0.0 | 93.2 |
| PSeSe46 | LUCO | 0.0 | 8.6 | 84.2 | PTeTe24 | LUCO | 0.0 | 2.0 | 91.4 |
| PSeSe46p | HOCO | 0.0 | 0.0 | 96.9 | PTeTe24p | HOCO | 0.0 | 0.0 | 97.5 |
| PSeSe46p | LUCO | 0.0 | 0.0 | 95.6 | PTeTe24p | LUCO | 0.0 | 0.0 | 97.9 |
| PSeTe24 | HOCO | 0.0 | 0.0 | 94.0 | PTeTe26 | HOCO | 0.0 | 7.7 | 84.1 |
| PSeTe24 | LUCO | 0.0 | 1.4 | 92.3 | PTeTe26 | LUCO | 26.2 | 37.0 | 28.3 |
| PSeTe24p | HOCO | 0.0 | 0.0 | 97.5 | PTeTe26p | HOCO | 0.0 | 0.0 | 98.1 |
| PSeTe24p | LUCO | 0.0 | 0.0 | 97.7 | PTeTe26p | LUCO | 51.0 | 36.0 | 0.0 |
| PSeTe26 | HOCO | 0.0 | 3.3 | 87.9 | PTeTe36 | HOCO | 0.5 | 28.0 | 63.6 |
| PSeTe26 | LUCO | 0.0 | 3.5 | 90.1 | PTeTe36 | LUCO | 14.1 | 37.8 | 38.5 |
| PSeTe26p | HOCO | 0.0 | 0.0 | 97.3 | PTeTe36p | HOCO | 0.0 | 0.0 | 99.0 |
| PSeTe26p | LUCO | 0.0 | 0.0 | 98.0 | PTeTe36p | LUCO | 0.0 | 0.0 | 97.6 |
| PSeTe36 | HOCO | 0.6 | 24.0 | 66.7 | PTeTe46 | HOCO | 5.6 | 19.2 | 68.5 |
| PSeTe36 | LUCO | 2.5 | 29.9 | 57.8 | PTeTe46 | LUCO | 0.0 | 27.5 | 65.2 |
| PSeTe36p | HOCO | 0.0 | 0.0 | 98.9 | PTeTe46p | HOCO | 0.0 | 0.0 | 95.7 |
| PSeTe36p | LUCO | 0.0 | 0.0 | 97.6 | PTeTe46p | LUCO | 0.0 | 0.0 | 97.5 |

**Table S2.** Some properties of frontier orbitals of the polymers (X, Y are heteroatoms of left monomer and X', Y' are heteroatoms of right monomer in the cell given in Fig. 5, the suffix p indicates planar form.)

| Polymer | Orbital | E (eV) | $E_g$ (eV) | s % | p % | d % | ODI | X % | Y % | X' % | Y' % |
|---|---|---|---|---|---|---|---|---|---|---|---|
| PSeSe24 | HOCO | -4.87 | | 0.01 | 98.22 | 1.76 | 11.04 | 0.44 | 3.53 | 0.44 | 3.53 |
| PSeSe24 | LUCO | -3.22 | 1.65 | 0.03 | 98.23 | 1.74 | 7.71 | 8.48 | 2.39 | 8.48 | 2.39 |
| PSeSe24p | HOCO | -4.85 | | 0.00 | 98.24 | 1.76 | 11.10 | 0.40 | 3.52 | 0.40 | 3.52 |
| PSeSe24p | LUCO | -3.23 | 1.63 | 0.00 | 98.26 | 1.74 | 7.74 | 8.46 | 2.28 | 8.45 | 2.28 |
| PSeSe26 | HOCO | -5.39 | | 0.10 | 98.65 | 1.24 | 10.14 | 3.79 | 5.64 | 3.80 | 5.63 |
| PSeSe26 | LUCO | -2.35 | 3.04 | 0.01 | 98.46 | 1.53 | 11.32 | 8.73 | 0.07 | 8.73 | 0.07 |
| PSeSe26p | HOCO | -5.40 | | 0.00 | 98.76 | 1.24 | 10.53 | 3.69 | 4.94 | 3.69 | 4.94 |
| PSeSe26p | LUCO | -2.39 | 3.01 | 0.00 | 98.49 | 1.51 | 11.50 | 8.65 | 0.08 | 8.65 | 0.08 |
| PSeSe36 | HOCO | -5.31 | | 0.33 | 98.52 | 1.14 | 10.40 | 1.31 | 16.97 | 1.32 | 16.95 |
| PSeSe36 | LUCO | -1.81 | 3.50 | 0.50 | 98.15 | 1.35 | 9.69 | 6.95 | 1.08 | 6.95 | 1.07 |
| PSeSe36p | HOCO | -5.32 | | 0.00 | 98.95 | 1.06 | 11.27 | 0.12 | 17.99 | 0.12 | 17.98 |
| PSeSe36p | LUCO | -2.28 | 3.04 | 0.00 | 98.75 | 1.25 | 10.21 | 6.04 | 0.54 | 6.04 | 0.54 |
| PSeSe46 | HOCO | -4.49 | | 0.00 | 98.34 | 1.66 | 8.79 | 0.63 | 8.54 | 0.63 | 8.54 |
| PSeSe46 | LUCO | -3.27 | 1.22 | 0.58 | 97.58 | 1.84 | 8.86 | 9.98 | 0.60 | 9.98 | 0.60 |
| PSeSe46p | HOCO | -4.26 | | 0.00 | 98.23 | 1.77 | 9.47 | 0.33 | 7.38 | 0.33 | 7.38 |
| PSeSe46p | LUCO | -3.48 | 0.78 | 0.00 | 98.29 | 1.71 | 10.61 | 10.89 | 1.09 | 10.88 | 1.10 |
| PSeTe24 | HOCO | -4.83 | | 0.12 | 98.22 | 1.66 | 10.42 | 0.47 | 5.06 | 0.46 | 5.06 |
| PSeTe24 | LUCO | -3.21 | 1.63 | 0.13 | 98.18 | 1.69 | 7.56 | 8.09 | 3.16 | 8.09 | 3.17 |
| PSeTe24p | HOCO | -4.77 | | 0.00 | 98.33 | 1.67 | 10.52 | 0.27 | 5.21 | 0.27 | 5.21 |
| PSeTe24p | LUCO | -3.22 | 1.54 | 0.00 | 98.28 | 1.72 | 7.76 | 8.09 | 2.12 | 8.08 | 2.12 |
| PSeTe26 | HOCO | -5.31 | | 0.17 | 98.68 | 1.14 | 9.65 | 3.36 | 9.12 | 3.36 | 9.12 |
| PSeTe26 | LUCO | -2.23 | 3.08 | 0.21 | 98.28 | 1.51 | 11.07 | 8.38 | 0.39 | 8.38 | 0.39 |
| PSeTe26p | HOCO | -5.35 | | 0.00 | 98.84 | 1.17 | 10.27 | 3.21 | 6.92 | 3.22 | 6.92 |
| PSeTe26p | LUCO | -2.31 | 3.03 | 0.00 | 98.53 | 1.47 | 11.48 | 8.54 | 0.14 | 8.54 | 0.14 |
| PSeTe36 | HOCO | -5.19 | | 0.35 | 98.66 | 0.98 | 12.52 | 1.23 | 21.26 | 1.23 | 21.26 |
| PSeTe36 | LUCO | -1.66 | 3.53 | 1.07 | 97.67 | 1.25 | 8.85 | 7.40 | 3.53 | 7.39 | 3.51 |
| PSeTe36p | HOCO | -5.24 | | 0.00 | 99.13 | 0.87 | 13.60 | 0.04 | 22.45 | 0.04 | 22.47 |
| PSeTe36p | LUCO | -2.19 | 3.05 | 0.00 | 98.77 | 1.23 | 10.21 | 5.95 | 0.41 | 5.95 | 0.41 |
| PSeTe46 | HOCO | -4.63 | | 0.01 | 98.51 | 1.48 | 8.89 | 1.03 | 10.95 | 1.03 | 10.95 |
| PSeTe46 | LUCO | -3.12 | 1.51 | 0.78 | 97.35 | 1.87 | 8.36 | 8.64 | 1.54 | 8.64 | 1.54 |
| PSeTe46p | HOCO | -4.30 | | 0.00 | 98.33 | 1.67 | 9.30 | 0.21 | 8.81 | 0.21 | 8.81 |
| PSeTe46p | LUCO | -3.39 | 0.91 | 0.00 | 98.31 | 1.69 | 10.22 | 10.60 | 1.32 | 10.60 | 1.32 |

**Table S2. (cont'd)** Some properties of frontier orbitals of the polymers (X, Y are heteroatoms of left monomer and X', Y' are heteroatoms of right monomer in the cell given in Fig. 5, the suffix p indicates planar form.)

| Polymer | Orbital | E (eV) | $E_g$ (eV) | s % | p % | d % | ODI | X % | Y % | X' % | Y' % |
|---|---|---|---|---|---|---|---|---|---|---|---|
| PTeSe24 | HOCO | -4.76 | | 0.06 | 98.20 | 1.74 | 11.04 | 0.56 | 3.68 | 0.57 | 3.67 |
| PTeSe24 | LUCO | -3.25 | 1.51 | 0.06 | 98.28 | 1.66 | 7.78 | 9.12 | 2.36 | 9.12 | 2.35 |
| PTeSe24p | HOCO | -4.72 | | 0.00 | 98.26 | 1.74 | 11.16 | 0.44 | 3.70 | 0.44 | 3.72 |
| PTeSe24p | LUCO | -3.27 | 1.46 | 0.00 | 98.33 | 1.67 | 7.82 | 8.99 | 2.10 | 8.99 | 2.10 |
| PTeSe26 | HOCO | -5.26 | | 0.16 | 98.64 | 1.19 | 9.37 | 5.65 | 5.67 | 5.66 | 5.66 |
| PTeSe26 | LUCO | -2.34 | 2.92 | -0.03 | 98.58 | 1.46 | 11.10 | 9.65 | 0.20 | 9.65 | 0.20 |
| PTeSe26p | HOCO | -5.27 | | 0.00 | 98.80 | 1.20 | 9.98 | 5.25 | 4.59 | 5.25 | 4.59 |
| PTeSe26p | LUCO | -2.39 | 2.88 | 0.00 | 98.54 | 1.46 | 11.53 | 8.65 | 0.13 | 8.65 | 0.13 |
| PTeSe36 | HOCO | -5.19 | | 0.39 | 98.50 | 1.11 | 9.86 | 2.57 | 16.43 | 2.57 | 16.43 |
| PTeSe36 | LUCO | -1.79 | 3.40 | 0.93 | 97.88 | 1.19 | 9.25 | 10.37 | 1.55 | 10.37 | 1.55 |
| PTeSe36p | HOCO | -5.26 | | 0.00 | 98.97 | 1.04 | 11.49 | 0.10 | 18.60 | 0.10 | 18.59 |
| PTeSe36p | LUCO | -2.34 | 2.92 | 0.00 | 98.78 | 1.22 | 10.08 | 5.69 | 0.55 | 5.69 | 0.55 |
| PTeSe46 | HOCO | -4.72 | | 0.06 | 98.42 | 1.51 | 8.69 | 2.44 | 8.63 | 2.44 | 8.64 |
| PTeSe46 | LUCO | -2.93 | 1.78 | 0.43 | 97.68 | 1.89 | 8.94 | 10.04 | 0.39 | 10.05 | 0.39 |
| PTeSe46p | HOCO | -4.25 | | 0.00 | 98.40 | 1.60 | 10.39 | 12.42 | 0.21 | 12.42 | 0.21 |
| PTeSe46p | LUCO | -3.50 | 0.75 | 0.00 | 98.27 | 1.73 | 11.03 | 2.18 | 7.10 | 2.18 | 7.10 |
| PTeTe24 | HOCO | -4.74 | | 0.20 | 98.15 | 1.65 | 10.47 | 0.62 | 4.96 | 0.60 | 4.96 |
| PTeTe24 | LUCO | -3.28 | 1.46 | 0.11 | 98.27 | 1.62 | 7.62 | 8.78 | 3.08 | 8.77 | 3.13 |
| PTeTe24p | HOCO | -4.67 | | 0.00 | 98.35 | 1.65 | 10.44 | 0.28 | 5.46 | 0.28 | 5.46 |
| PTeTe24p | LUCO | -3.29 | 1.37 | 0.00 | 98.34 | 1.66 | 7.82 | 8.65 | 1.86 | 8.65 | 1.86 |
| PTeTe26 | HOCO | -5.19 | | 0.23 | 98.67 | 1.10 | 8.91 | 5.19 | 9.29 | 5.19 | 9.29 |
| PTeTe26 | LUCO | -2.26 | 2.94 | 2.93 | 96.22 | 0.84 | 10.20 | 17.32 | 9.18 | 17.32 | 9.19 |
| PTeTe26p | HOCO | -5.24 | | 0.00 | 98.87 | 1.13 | 9.75 | 4.63 | 6.38 | 4.63 | 6.38 |
| PTeTe26p | LUCO | -2.53 | 2.72 | 8.48 | 91.16 | 0.36 | 13.87 | 17.96 | 18.19 | 17.96 | 18.19 |
| PTeTe36 | HOCO | -5.07 | | 0.37 | 98.67 | 0.96 | 11.95 | 2.26 | 20.69 | 2.25 | 20.71 |
| PTeTe36 | LUCO | -1.71 | 3.36 | 2.46 | 96.59 | 0.94 | 8.79 | 13.59 | 5.94 | 13.76 | 5.98 |
| PTeTe36p | HOCO | -5.17 | | 0.00 | 99.15 | 0.85 | 14.03 | 0.02 | 23.19 | 0.02 | 23.18 |
| PTeTe36p | LUCO | -2.25 | 2.93 | 0.00 | 98.81 | 1.19 | 10.06 | 5.62 | 0.40 | 5.63 | 0.40 |
| PTeTe46 | HOCO | -4.84 | | 0.08 | 98.59 | 1.33 | 8.91 | 2.82 | 11.81 | 2.82 | 11.82 |
| PTeTe46 | LUCO | -2.77 | 2.07 | 0.42 | 97.67 | 1.90 | 8.70 | 9.21 | 1.09 | 9.21 | 1.09 |
| PTeTe46p | HOCO | -4.26 | | 0.00 | 98.30 | 1.70 | 10.43 | 0.71 | 7.29 | 0.70 | 7.29 |
| PTeTe46p | LUCO | -3.53 | 0.73 | 0.00 | 98.47 | 1.53 | 11.78 | 11.15 | 2.33 | 11.15 | 2.33 |

**Table S3.** The band gap, HOCO and LUCO values (in eV) calculated by B3PW91/SDD method.

| Band gap (eV) | | | |
|---|---|---|---|
| Y/X | Se | Te | Po |
| Se | 0.766 | 0.723 | 0.813 |
| Te | 0.885 | 0.717 | 0.663 |
| Po | 0.919 | 0.764 | 0.686 |
| HOCO (eV) | | | |
| Y/X | Se | Te | Po |
| Se | -4.358 | -4.366 | -4.330 |
| Te | -4.433 | -4.409 | -4.310 |
| Po | -4.333 | -4.331 | -4.246 |
| LUCO (eV) | | | |
| Y/X | Se | Te | Po |
| Se | -3.592 | -3.644 | -3.517 |
| Te | -3.548 | -3.693 | -3.647 |
| Po | -3.413 | -3.567 | -3.561 |

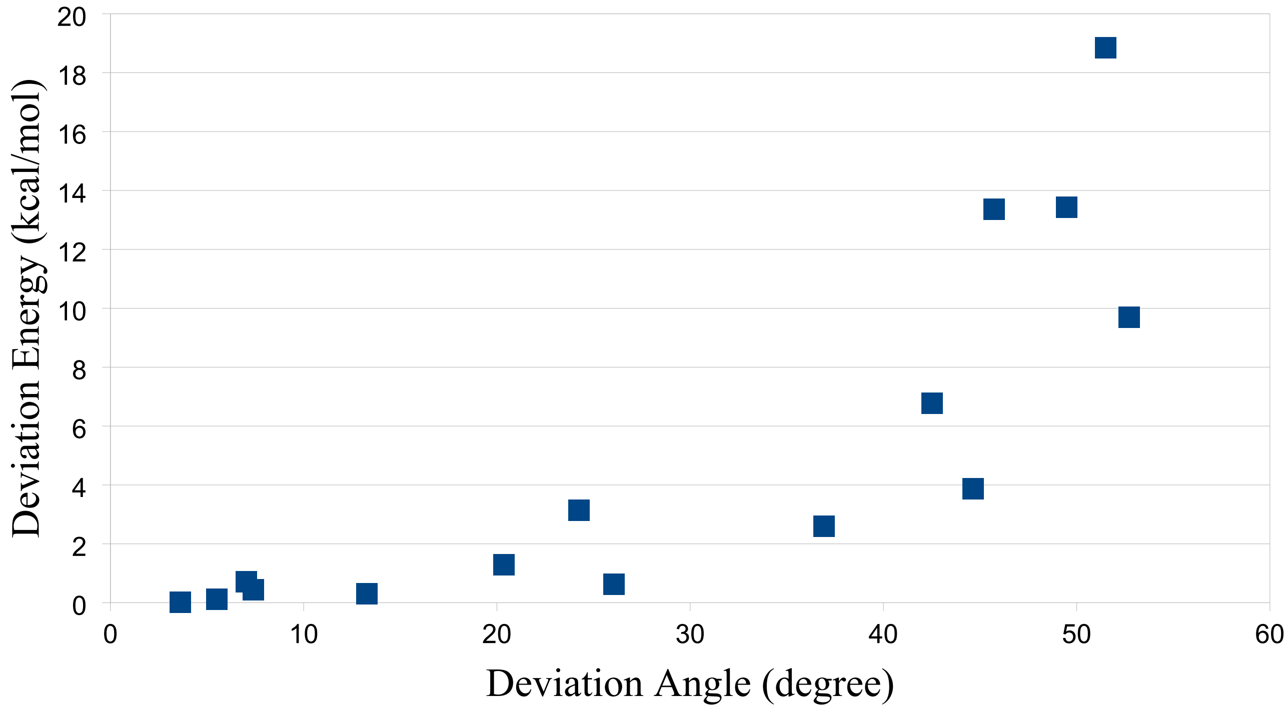


**Figure S1.** The deviation energies ($E_{planar}$-$E_{nr}$) versus the deviation angle (from 180$^{o}$).

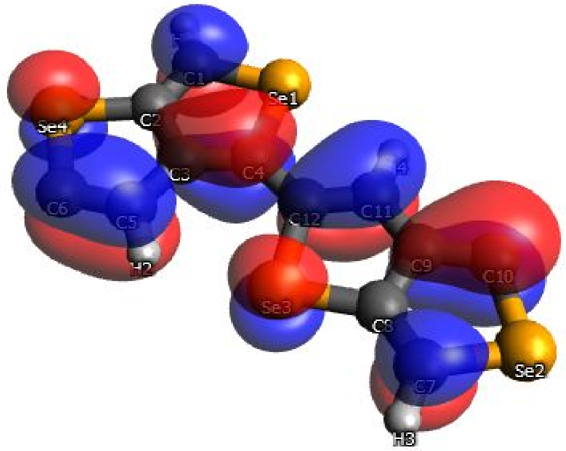

**PSeSe24**

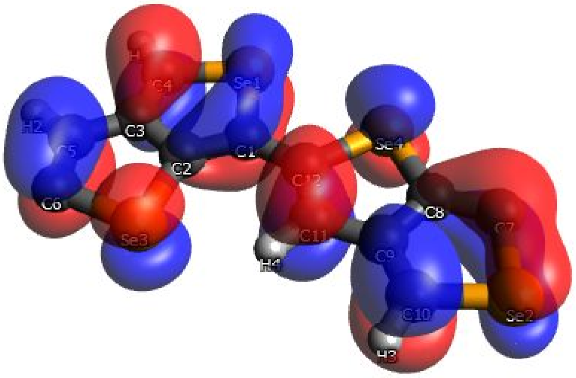

**PSeSe26**

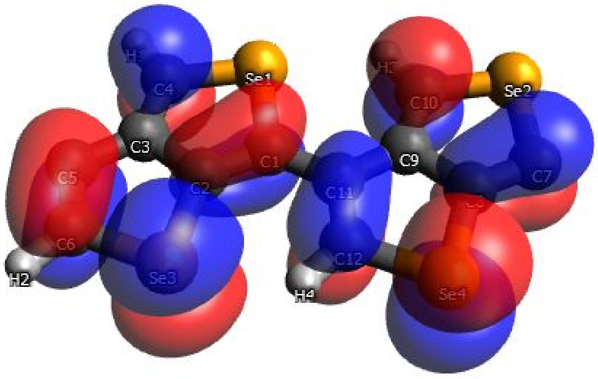

**PSeSe36**

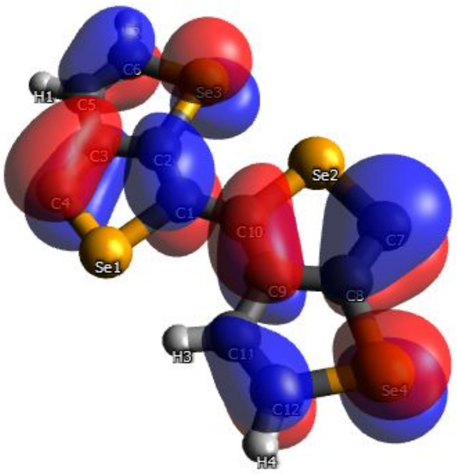

**PSeSe46**

**Figure S2.** HOCOs of PSeSe for planar geometry.

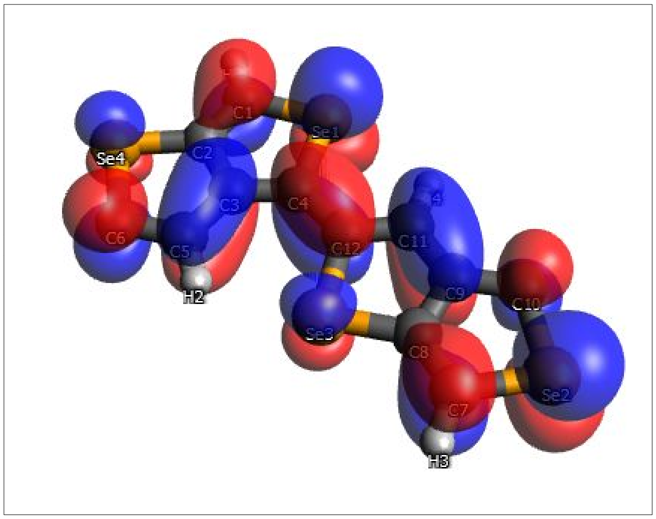

**PSeSe24**

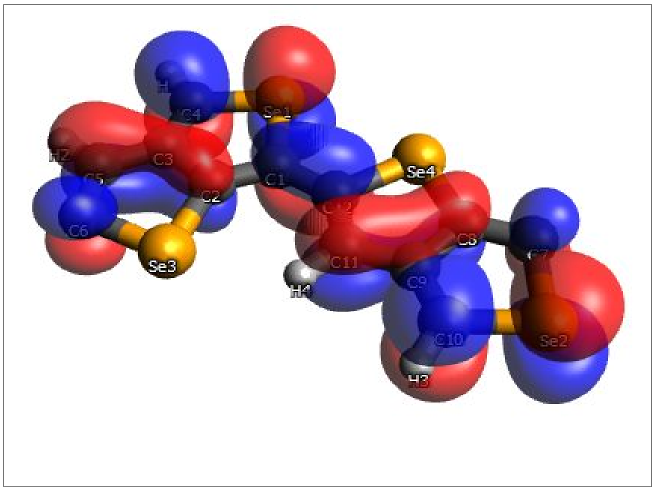

**PSeSe26**

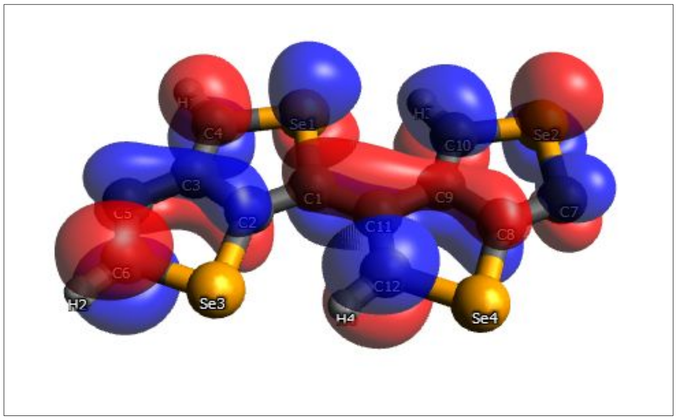

**PSeSe36**

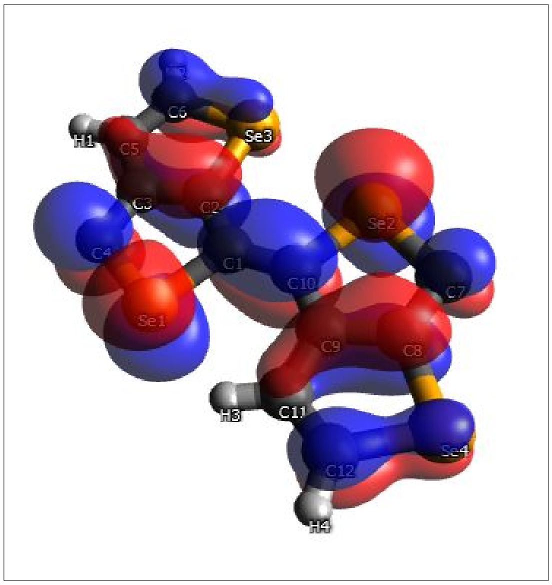

**PSeSe46**

**Figure S3.** LUCOs of PSeSe for planar geometry.
